# Genomic reconstruction of σ^54^ regulons in *Clostridiales*

**DOI:** 10.1186/s12864-019-5918-4

**Published:** 2019-07-09

**Authors:** Xiaoqun Nie, Wenyue Dong, Chen Yang

**Affiliations:** 1 CAS-Key Laboratory of Synthetic Biology, CAS Center for Excellence in Molecular Plant Sciences, Shanghai Institute of Plant Physiology and Ecology, Chinese Academy of Sciences, Shanghai, 200032 China; 2 University of Chinese Academy of Sciences, Beijing, China

**Keywords:** σ^54^, Enhancer binding protein, Transcriptional regulation, *Clostridium*, Comparative genomics

## Abstract

**Background:**

The σ^54^ factor controls unique promoters and interacts with a specialized activator (enhancer binding proteins [EBP]) for transcription initiation. Although σ^54^ is present in many *Clostridiales* species that have great importance in human health and biotechnological applications, the cellular processes controlled by σ^54^ remain unknown.

**Results:**

For systematic analysis of the regulatory functions of σ^54^, we performed comparative genomic reconstruction of transcriptional regulons of σ^54^ in 57 species from the *Clostridiales* order. The EBP-binding DNA motifs and regulated genes were identified for 263 EBPs that constitute 39 distinct groups. The reconstructed σ^54^ regulons contain the genes involved in fermentation and amino acid catabolism. The predicted σ^54^ binding sites in the genomes of *Clostridiales* spp. were verified by in vitro binding assays. To our knowledge, this is the first report about direct regulation of the Stickland reactions and butyrate and alcohols synthesis by σ^54^ and the respective EBPs. Considerable variations were demonstrated in the sizes and gene contents of reconstructed σ^54^ regulons between different *Clostridiales* species. It is proposed that σ^54^ controls butyrate and alcohols synthesis in solvent-producing species, regulates autotrophic metabolism in acetogenic species, and affects the toxin production in pathogenic species.

**Conclusions:**

This study reveals previously unrecognized functions of σ^54^ and provides novel insights into the regulation of fermentation and amino acid metabolism in *Clostridiales* species, which could have potential applications in guiding the treatment and efficient utilization of these species.

**Electronic supplementary material:**

The online version of this article (10.1186/s12864-019-5918-4) contains supplementary material, which is available to authorized users.

## Background

The sigma (σ) subunit is required for promoter recognition and initiation of transcription by the bacterial RNA polymerase (RNAP). σ^54^ is unique in that it shares no detectable homology with any of the other known sigma factors (e.g., σ^70^) and binds to conserved − 12 and − 24 promoter elements [1]. The σ^54^-dependent transcription absolutely requires the presence of an activator that couples the energy generated from ATP hydrolysis to the isomerization of the RNA polymerase-σ^54^ closed complex [2]. These activators are usually called enhancer binding proteins (EBPs) and bind to upstream activator sequences (UAS) located upstream of the promoter. EBPs are modular proteins and generally consist of three domains [3, 4]. The regulatory domain has a role in signal perception and modulates the activity of the EBPs. The AAA^+^ (ATPase associated with cellular activities) domain is responsible for ATP hydrolysis and interaction with σ^54^. The DNA-binding domain enables recognition of specific UAS site. DNA looping is required for the activator to contact the closed complex and catalyze formation of the open promoter complex [5].

The σ^54^ regulons have been extensively studied in several model organisms. In *Escherichia coli*, σ^54^ was identified as a sigma factor for transcription of genes involved in the assimilation of ammonia and glutamate under conditions of nitrogen limitation [6]. This σ^54^-dependent transcription requires the activator NtrC that is phosphorylated by the sensor kinase NtrB in response to the nitrogen status of the cell [7]. The involvement of σ^54^ in flagellar biosynthesis, formate metabolism, and phage shock response was also found in *E. coli*. It was considered that the physiological themes of the vast majority of σ^54^-dependent genes in *E. coli* may be related to nitrogen assimilation [8]. In many diazotrophic *Proteobacteria* such as *Azotobacter vinelandii*, transcription of the genes required for nitrogen fixation are dependent on σ^54^ [9]. In addition, other physiological functions such as catabolism of toluene and xylenes in *Pseudomonas putida* as well as utilization of levan and acetoin in *Bacillus subtilis* are also controlled by σ^54^ [10–12].

Organisms of the order *Clostridiales* are Gram-positive obligate anaerobes important in human health and physiology, the carbon cycle, and biotechnological applications [13, 14]. For example, *Clostridium beijerinckii*, *Clostridium acetobutylicum*, *Clostridium saccharobutylicum*, and *Clostridium saccharoperbutylacetonicum* can ferment carbohydrates and produce solvents [15]. The acetogenic *Clostridium ljungdahlii*, *Clostridium carboxidivorans*, *Clostridium autoethanogenum*, *Acetobacterium woodii* are able to fix CO_2_ or CO [16]. Several *Clostridiales* species are significant human pathogens, including *Clostridioides difficile* that is an important cause of diarrhea, *Clostridium botulinum*, *Clostridium tetani*, and *Clostridium perfringens* that are the etiological agents of botulism, tetanus, and gas gangrene, respectively [17]. On the other hand, some *Clostridiales* species are believed to have positive effect on human health, including *Clostridium butyricum* that is widely used as a probiotic and *Clostridium novyi* that has potential therapeutic uses in cancers [18, 19]. Recently, several *Clostridiales* species have been isolated from animal gut, including *Romboutsia ilealis* and *Romboutsia* sp. FRIFI, which are natural resident and key players in the small intestinal of animals [20].

Our previous study has identified some σ^54^-dependent genes in several *Clostridium* species, which are activated by the phosphoenolpyruvate-dependent phosphotransferase system regulation domain (PRD)-containing EBPs and involved in utilization of β-glucosides, fructose/levan, pentitols, and glucosamine/fructosamine [21]. However, the regulatory functions of the majority of the EBPs in *Clostridiales* species remain unknown. Our knowledge about the cellular processes controlled by σ^54^ in *Clostridiales* is limited, because the σ^54^ regulons have not been systematically analyzed in these organisms.

In this study, we used a comparative genomic approach to reconstruct σ^54^-dependent transcriptional regulons in 57 species from the *Clostridiales* order. We identified putative EBPs and their regulatory modules. The candidate targets of σ^54^ and 263 EBPs, which constitute 39 distinct EBP groups, were identified based on the recognition of the EBP-binding DNA motifs, candidate UAS sites, and conserved σ^54^ promoter elements. Some of the predicted σ^54^-dependent promoters upstream of putative target genes in the genomes of *Clostridium* spp. were validated by in vitro binding assays. Considerable variations were found in the sizes and gene contents of reconstructed σ^54^ regulons between different species. Based on the gene contents of the reconstructed regulons, novel functions of σ^54^ and the respective EBPs were identified, including direct regulation of the Stickland reactions and butyrate and alcohols synthesis.

## Results

### Repertoire of σ^54^ and EBPs in *Clostridiales*

**Fig. 1:**
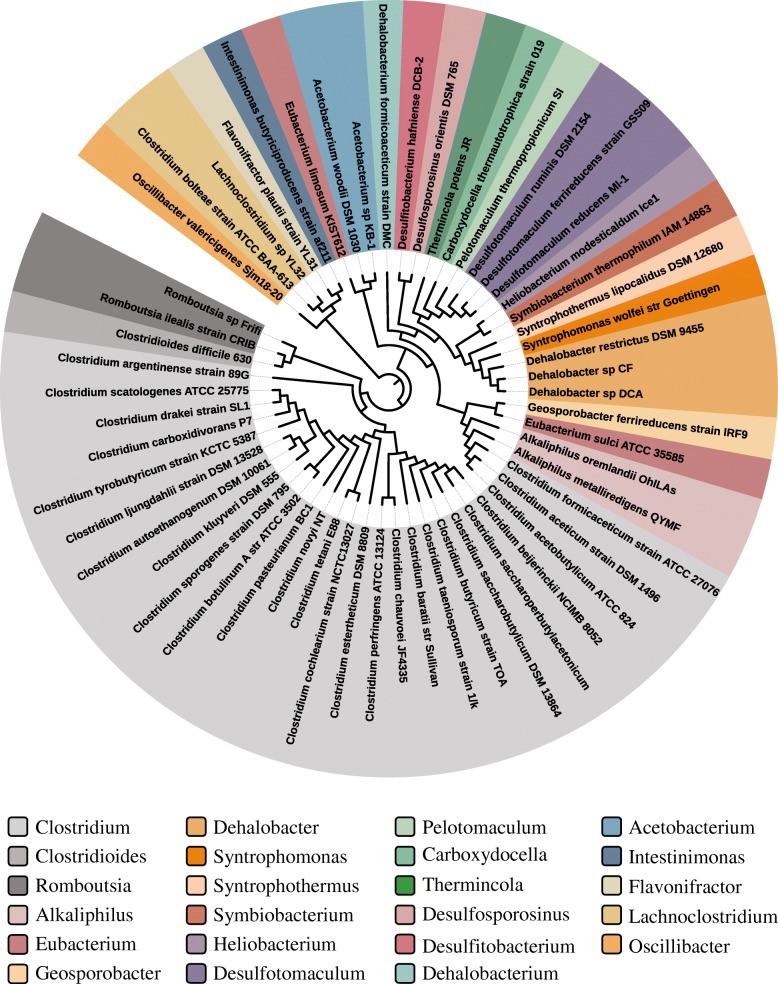
The maximum-likelihood phylogenetic tree of σ^54^ (SigL) in 57 species from 23 genera of *Clostridiales*

For identification of σ^54^ (SigL) in *Clostridiales* species, orthologs of SigL from *B. subtilis* was searched in 124 completely sequenced genomes. The SigL orthologs were found in 57 genomes from 23 genera including *Clostridium*, *Clostridioides*, *Eubacterium*, *Acetobacterium*, and *Dehalobacter* (Fig. 1; Additional file 1: Table S1). Each of these genomes has a single copy of *sigL*. Among the 23 genera, *Clostridium* genus has the largest number of SigL orthologs. The SigL was identified in 26 genomes of *Clostridium* genus, including *C. beijerinckii*, *C. acetobutylicum*, *C. ljungdahlii*, *C. botulinum*, and *C. tetani*. However, some species in *Clostridium* genus such as cellulolytic *Clostridium cellulovorans* lack a SigL ortholog. In each of the other genera, SigL was found in only one to three species. Thus, σ^54^ is widely present among *Clostridiales*. However, its presence seemingly had no obvious correlation with the phylogeny of *Clostridiales* species.

For identification of EBPs that are σ^54^-dependent transcriptional activators, the experimentally characterized EBPs proteins including NtrC from *E. coli*, AcoR and LevR from *B. subtilis* were used for homologous search in the *Clostridiales* species that have the σ^54^-encoding gene. A total of 490 EBPs were identified in 57 *Clostridiales* species. The presence of the peptide motif ‘GAFTGA’ was checked in the identified EBPs, which is necessary for the interaction with σ^54^ [22]. An exact GAFTGA sequence was observed in 355 out of 490 EBPs (Additional file 1: Table S2). The other 100 EBPs possess some variants of the motif (e.g., GSFTGA, GAYTGA, GAFSGA), which still allow the EBP to activate σ^54^-dependent transcription [3]. Our regulon reconstruction results (see below) also suggested that these variants do not prevent the respective EBP from activating σ^54^ promoters.

Each of the analyzed *Clostridiales* species possesses one to thirty-five EBPs (Additional file 1: Table S1). The number of EBPs is highly variable between different species. A significant positive correction was observed between the EBPs number and the genome size with the spearman correlation test (*p* < 0.0001) (Additional file 1: Table S1), similar to the results of the previous report [6].

The majority (431 out of 490; 77%) of the identified EBPs in *Clostridiales* consist of a central AAA^+^ domain, an N-terminal regulatory domain, and a DNA-binding domain (DBD) at the C-terminus (Additional file 1: Table S2). Forty-eight EBPs possess the PRD domain at the C-terminus [21]. The remaining 11 EBPs lack the N-terminal regulatory domain, which is similar to PspF from *E. coli* [23].

**Fig. 2:**
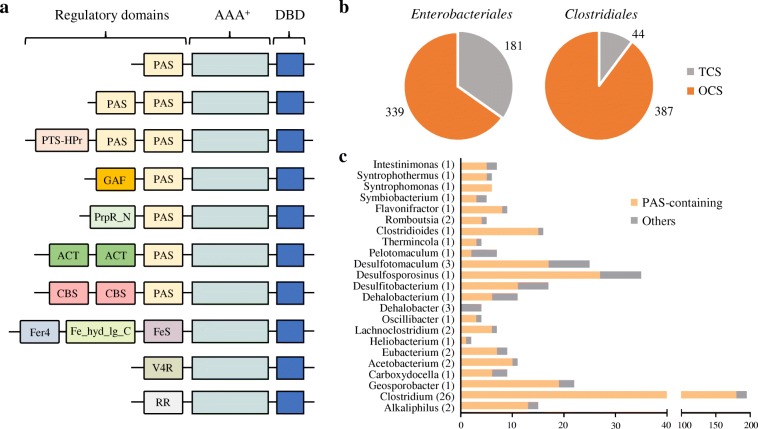
Domain organization of EBPs in *Clostridiales*. **a** The domain architecture of EBPs. PAS, Per-Arnt-Sim domain; GAF, cyclic GMP-specific phosphodiesterases, adenylyl cyclases and FhlA; PTS-HPr, PTS system histidine phosphocarrier protein HPr-like; PrpR_N, N-terminal domain of Propionate catabolism activator; ACT, aspartokinase-chorismate mutase-TyrA; CBS, cystathinoine β-synthase domain; Fer4, 4Fe-4S binding domain; Fe_hyd_lg_C, iron only hydrogenase large subunit, C-terminal domain; FeS, Fe-S cluster; V4R, vinyl 4 reductase domain; RR, response regulator domain. **b** Distribution of TCS and OCS-type EBPs in *Enterobacteriales* and *Clostridiales*. **c** Distribution of PAS-containing EBPs in 23 genera of *Clostridiales*

The N-terminal regulatory domain, which responds to environmental signals and modulates EBP activity [24], is not well conserved between the identified EBPs in *Clostridiales* species (Fig. 2a). A variety of domains were found in the regulatory region of the 431 EBPs, including PAS domains (Pfam clan accession no. CL0183), GAF domains (CL0161), PTS-HPr domains (PF00381), PrpR_N domains (PF06506), ACT domains (PF01842), CBS domains (PF00571), Fer4 domains (PF00037), Fe_hyd_lg_C domains (PF02906), FeS domains (PF04060), V4R domains (PF02830), and response regulator (RR) domains (Fig. 2a). Most of these domains lack transmembrane regions, suggesting that the EBPs in *Clostridiales* mainly sense intracellular signals. Interestingly, only 44 EBPs (~ 10%) have the RR domains that are part of two-component systems (TCSs) and phosphorylated by specific sensor kinases (Fig. 2b). The other 387 EBPs are one-component regulatory systems (OCSs) containing a regulatory domain that directly binds small effector molecules. This is different from the situation in *Enterobacteriales*, in which a larger fraction of EBPs (~ 35%) has the RR domains (Fig. 2b). This result indicates that the EBPs in *Clostridiales* respond to environmental signals mainly through ligand binding rather than phosphorylation of the N-terminal regulatory domain.

Almost all the OCS-type EBPs (357 out of 387) contain the PAS domains, which can bind various cofactors and ligands and are often found in signaling proteins [25, 26]. These PAS domain-containing EBPs are widely distributed in nearly all the analyzed *Clostridiales* species (Fig. 2c). The PAS domains are present as single domain, in two copies, or adjacent to other domains on the same EBP (Fig. 2a). This suggests that the PAS domains play an important role in signal sensing or transduction, thereby modulating the activity of a large number of EBPs in *Clostridiales* species.

### Reconstruction of regulons of σ^54^-dependent transcriptional activators in *Clostridiales*

To reconstruct transcriptional regulons for the repertoire of the EBPs in *Clostridiales*, we used the integrative comparative genomics approach that combines identification of candidate DNA binding sites of EBPs and σ^54^ with cross-genomic comparison of regulons (see Methods for details). The DNA-binding domain of 335 EBPs in *Clostridiales* contains a Fis-type helix-turn-helix (HTH) motif (Pfam accession no. PF02954), which allows recognition of specific EBP binding sites (UAS sites). We identified the conserved UAS motifs and reconstructed the regulons for 263 EBPs that constitute 39 groups with two or more orthologs. The remaining EBPs lack orthologs in the sequenced *Clostridales* genomes, thus comparative genomics approach cannot be applied reliably. Among the 39 orthologous groups of EBPs, four groups are PRD-containing EBPs, for which the UAS motifs have been identified previously [21]. We named the individual EBP groups based on the functional content analysis of the reconstructed regulons as described below.

**Fig. 3:**
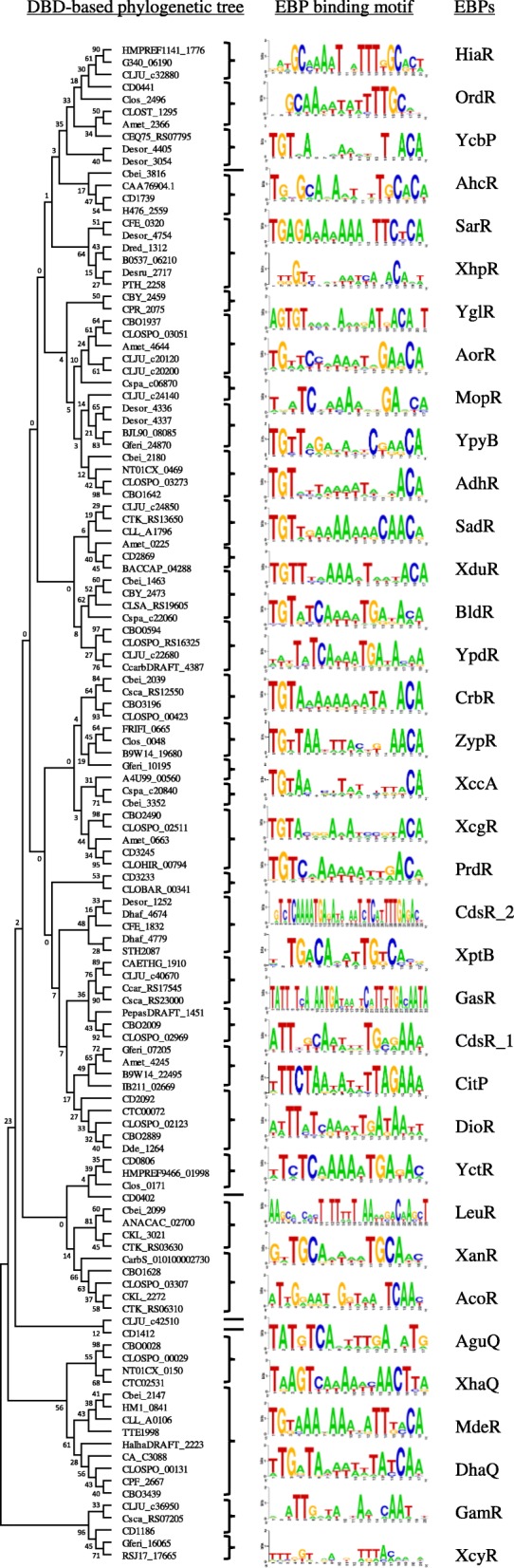
Phylogenetic tree of DNA-binding domains of EBPs and identified EBP-binding DNA motifs in *Clostridiales*

The identified UAS motif for each orthologous group of EBPs is shown in Fig. 3 and Additional file 1: Table S3. The motifs for fifteen groups including YcbP, AhcR, XhpR, AorR, YpyB, AdhR, SadR, XduR, BldR, CrbR, ZypR, XccA, XcgR, PrdR, MdeR, consist of two inverted repeats TGT and ACA separated by 10–12-bp spacer, which is similar to the UAS motifs for the well-characterized EBPs such as FhlA in *E. coli* [27] and NifA in *Klebsiella pneumoniae* [28]. Comparison of all the identified UAS motifs using TOMTOM [29] found similarity in the motifs for the other 6 groups (i.e., SarR, YglR, XptB,AguQ, XhaQ, DhaR). However, distinct DNA motifs were found for the remaining 15 groups (i.e., HiaR, OrdR, MopR, YpdR, CdsR1/2, GasR, CitP, DioR, YctR, LeuR, XanR, AcoR, GamR, XcyR). Similarity of the UAS motifs is consistent with the similarity of the DNA-binding domains of EBPs (Fig. 3).

All the candidate UAS sites for 263 EBPs were detected using these obtained DNA motifs. Moreover, we used the σ^54^ promoter sequence motif with the consensus TTGGCATNNNNNTTGCT to search for candidate σ^54^ binding sites in 57 *Clostridiales* genomes [30]. The details about the target operons of individual EBPs, and their upstream UAS sites and σ^54^-binding sites are listed in Additional file 1: Table S3.

**Fig. 4:**
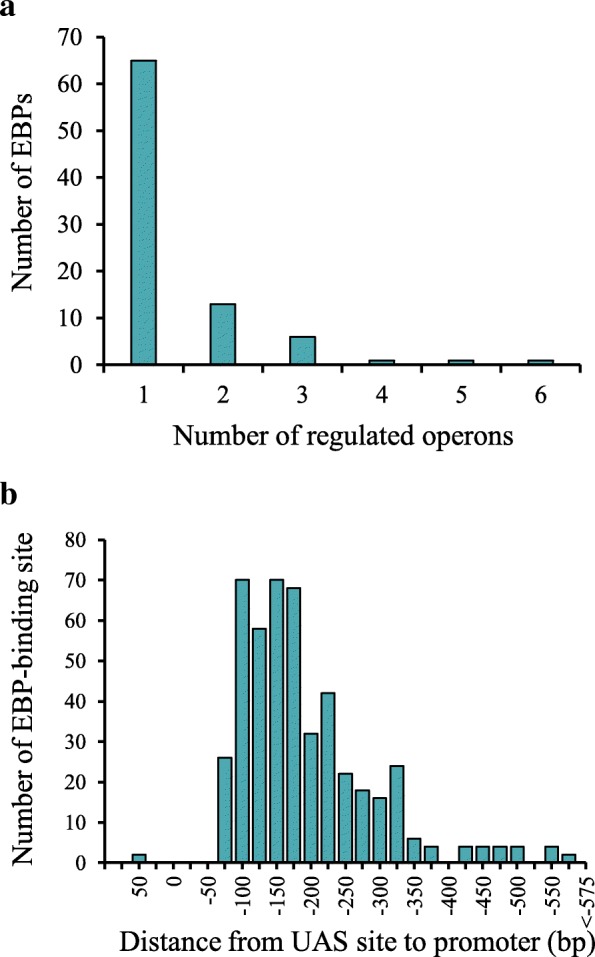
Distribution of **a** EBPs regulons sizes and **b** distances between UAS site and σ^54^ promoter

The majority of the EBPs (170 out of 263; 65%) was found to control only one target operon (Fig. 4a). The rest 93 EBPs have larger regulons with two to six operons. Most of the predicted target operons are co-localized with the respective EBP-encoding genes on the chromosome. This is coincident with previous findings that the EBP-encoding genes are usually close or adjacent to their target genes [4, 31]. However, 38 EBPs belonging to 12 orthologous groups were found not positionally clustered with the regulated genes (Additional file 1: Table S3). For 32 orthologous groups comprising of 198 EBPs, the target operons are preceded by multiple UAS sites. The σ^54^ binding sites were identified within the promoter regions of all the candidate target operons of EBPs. Most of the detected UAS sites are situated in the upstream of the candidate σ^54^ promoter at a distance of 100–250 bp (Fig. 4b).

### Functional content of reconstructed σ^54^ regulons in *Clostridiales*

**Fig. 5:**
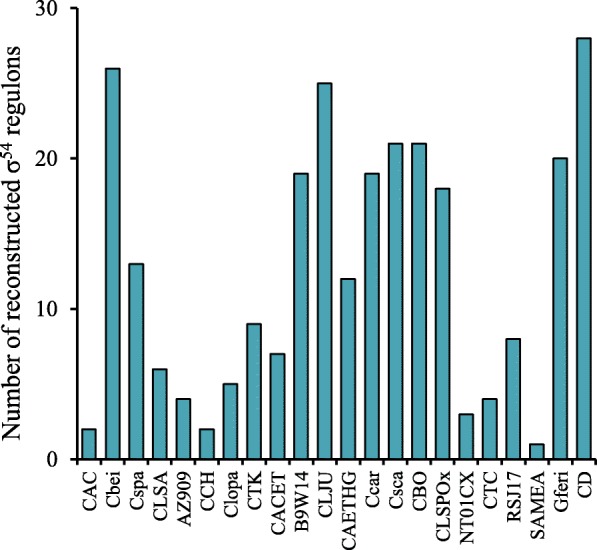
The sizes of reconstructed σ^54^ regulons in representative species of *Clostridiales*. CAC, *C. acetobutylicum*; Cbei, *C. beijerinckii*; Cspa, *C. saccharoperbutylacetonicum*; CLSA, *C. saccharobutylicum*; ZA909, *C. butyricum*; CCH, *Clostridium chauvoei*; Clopa, *Clostridium pasteurianum*; CTK, *Clostridium tyrobutyricum*; CACET, *C. aceticum*; B9W14, *Clostridium drakei*; CLJU, *C. ljungdahlii*; CAETHG, *C. autoethanogenum*; Ccar, *C. carboxidivorans*; Csca, *C. scatologenes*; CBO, *C. botulinum*; CLSPOx, *C. sporogenes*; NT01CX, *C. novyi*; CTC, *C. tetani*; RSJ17, *C. argentinense*; SAMEA, *Clostridium cochlearium*; Gferi, *G. ferrireducens*; CD, *C. difficile*

We tentatively predicted possible biological functions of σ^54^ and EBPs by assessing the functional context of the target operons. We were able to predict the functions for 31 out of 39 orthologous EBP groups (Table 1). These EBPs were named based on the functional content analysis of the target genes. For the remaining eight groups, the functions of the target genes are unknown. We observed that the sizes of reconstructed σ^54^ regulons vary significantly in different *Clostridiales* species (Fig. 5). For instance, the σ^54^ regulon contains 26 operons in *C. beijerinckii*, whereas in *C. acetobutylicum* only two operons are σ^54^-controlled. The total number of regulons per genome varies from one to twenty-eight. Not a single operon is potentially regulated by σ^54^ in all the analyzed species.

**Table 1:** Reconstructed EBP regulons of *Clostridiales*

EBP	Target operon	Function	Distribution
AcoR	*acoABCL*	Acetoin catabolism	*Clostridium*
AdhR	*adhA*	Alcohol synthesis	*Clostridium*
AguQ	*aguDA*, *aguBC*, *aguQT*	Agmatine metabolism	*Clostridium*
AhcR	*atoE-hbd-cotX*	Butyrate/Butanol synthesis	*Clostridium*
AorR	*aor-moaD*	Alcohol synthesis	*Clostridium*, *Geosporobacter*
AtlR	*atlEFG*, *atlD-tkt-rpi-araD-manB*	Pentitol catabolism	*Clostridioides*, *Clostridium*, *Geosporobacter, Lachnoclostridium*
BldR	*butA*	2,3-Butanediol synthesis	*Clostridium*
CelR	*celCA-bglB-celB*, *celFB*	Cellobiose catabolism	*Alkaliphilus*, *Clostridium*
CitP	*citA*	Transporter	*Alkaliphilus*, *Clostridium*, *Geosporobacter*
CrbR	*crt-hbd-thl-maoC-bcd-etfAB*, *cotX-gntT*	Butyrate/Butanol synthesis	*Clostridium*
DhaQ	*dhaKLM*, *ptsI*	Dihydroxyacetone metabolism	*Acetobacterium*, *Clostridium*, *Geosporobacter*
DioR	*dpaL-pyrC-argE*, *tdcF*, *yqeB*, *pbuX, ygeW-ygfK-ssnA-ygeY*, *dioR,* *yqeC-ygfJ-yqeB-xdhC-hyp-fepDCB*	Unknown	*Clostridioides*, *Clostridium*, *Desulfotomaculum*
GamR	*puuDT*	Amino acid metabolism	*Clostridioides*, *Clostridium*, *Romboutsia*
GasR	*puuXP*	Amino acid metabolism	*Clostridium*
GfrR	*gfrABCDEF*	Glucosamine/fructosamine catabolism	*Clostridioides*, *Clostridium*
HiaR	*hiaL-hyp-hutH*	Histidine catabolism	*Clostridium*
CdsR	*cdsB*	Cysteine catabolism	*Clostridium*, *Clostridioides*
LeuR	*hadAIBC-acdB-etfBA*	Leucine catabolism	*Clostridioides*
LevR	*levDEFG-sacC*	Levan catabolism	*Clostridium*
MdeR	*mdeA-metT*	Methionine catabolism	*Clostridium*
MopR	*mopR*, *mop*, *hyp-moeA*	Unknown	*Clostridium*
OrdR	*ord-ortBA-oraSEF-orr-nhaC*	Ornithine catabolism	*Clostridioides*, *Clostridium*, *Geosporobacter*, *Romboutsia*
PrdR	*prdABDEF*, *prdC*	Proline catabolism	*Alkaliphilus*, *Clostridioides*, *Clostridium*, *Geosporobacter*
SadR	*sadh*	Alcohol synthesis	*Clostridium*
SarR	*grdGF*	Sarcosine metabolism	*Clostridioides*
XanR	*abfD-nifU-hyp-ach-golB*, *acp-caiC-luxEC*	Butyrate/Butanol synthesis	*Clostridium*
XcaA	*thl*	Unknown	*Clostridium*, *Geosporobacter*
YcbP	*phyL-uroD-eamA*	Unknown	*Dehalobacterium*, *Desulfosporosinus*
XcgR	*cotY-gntT-bcd-etfAB*	Butyrate/Butanol synthesis	*Clostridium*, *Geosporobacter*
XcyR	*malY-pepT-hyp*	Peptide degradation	*Clostridioides*, *Clostridium*, *Flavonifractor, Geosporobacter*, *Lachnoclostridium*
XduR	*kdgT-uxaA*	Pectin degradation	*Alkaliphilus*, *Clostridioides*, *Clostridium*, *Geosporobacter*
XhaQ	*rhaT*	Transporter	*Clostridioides*
XhpR	*hyp-memP*	Unknown	*Desulfosporosinus*, *Carboxydocella*, *Desulfotomaculum*
XptB	*sulT*, *hyp*	Unknown	*Dehalobacterium*, *Desulfitobacterium*, *Desulfosporosinus, Carboxydocella*
YctR	*crt-gntT-caiB-bcd-etfAB*	Butyrate/Butanol synthesis	*Clostridioides*
YglR	*hyp-gltD*	Unknown	*Clostridium*
YpdR	*metM-memP-pepABC*, *fmdE-metM-hemABC*	Peptide degradation	*Clostridium*
YpyB	*pdc-tdh-hyp*	Unknown	*Clostridium*, *Desulfosporosinus*, *Geosporobacter*
ZypR	*tyrB-iorAB-butK*	Amino acid metabolism	*Clostridioides*, *Clostridium*, *Romboutsia*

**Fig. 6:**
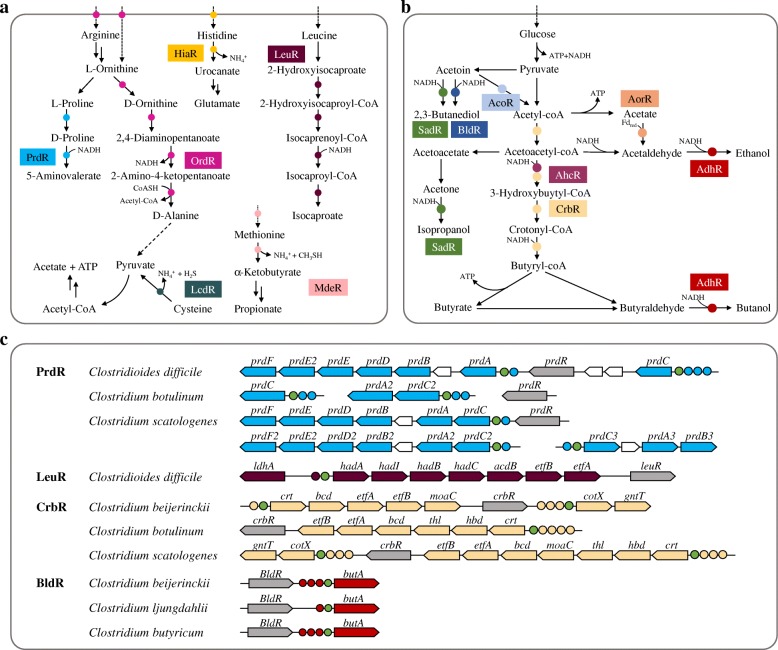
Metabolic context of the reconstructed σ^54^ regulons in *Clostridiales* species. EBPs and regulated genes are involved in **a** amino acid catabolism and **b** fermentation. Individual EBPs and corresponding target genes are shown by matching background colors. **c** Functional and genomic context of representative EBPs regulons. EBP-encoding genes are shown by gray arrows, and target genes (shown by arrows) from the same metabolic pathway are shown with the same color. EBP binding sites are indicated by circle with matching colors, and σ^54^ binding sites are marked by green circle

The reconstructed σ^54^ regulons control the metabolism in all of the analyzed *Clostridiales* species. The *acoABCL* operon involved in acetoin catabolism, which is σ^54^-dependent in *B. subtilis* [12], is present in the reconstructed clostridial σ^54^ regulons. The genes involved in transport of arginine/ornithine and histidine (i.e., *nhaC* and *hiaL*) are predicted to be σ^54^-dependent. The same function has been reported for the σ^54^ in *E. coli* [8], although the target genes are not orthologous. More importantly, we observed some members of the σ^54^ regulons in *Clostridiales*, which have not been described in any other bacteria. These operons are involved in fermentation and amino acid catabolism (Fig. 6), particularly in butyrate and alcohols synthesis and the Stickland reactions, as described in detail below.

### Regulation of the Stickland reactions

In *C. difficile* and some other related species, the reconstructed σ^54^ regulons contain the genes involved in amino acid metabolism, especially the Stickland reactions (Fig. 6a). The Stickland reactions couple the oxidation and reduction of amino acids to their corresponding organic acids, which serves as a primary source of energy generation in *Clostridium* species [32]. This process strongly influences the production of toxins in pathogenic clostridia [33, 34].

Proline is one of the most efficient electron acceptors in the Stickland reactions [35]. The *prdA*, *prdB*, *prdC*, *prdD*, *prdE*, and *prdF* genes, which are involved in reduction of proline to 5-aminovalerate, were predicted to be σ^54^-dependent in nine *Clostridiales* species (Table 1). The *prdABCDEF* operon is preceded by putative σ^54^ promoter and multiple UAS sites of PrdR in the genome of *C. difficile* (Fig. 6c). Consistently, a previous study has shown that PrdR activates the expression of *prd* operon and negatively affects the expression of toxin gene in *C. difficile* [36]. We predicted that the genes involved in proline reduction, which are either clustered or stand-alone on the genome, are regulated by σ^54^ and PrdR in eight other species including *C. botulinum, Clostridium scatologenes, Clostridium sporogenes, Clostridium formicaceticum, Alkaliphilus metalliredigenes, Clostridium argentinense, Geosporobacter ferrireducens, Alkaliphilus oremlandii* (Additional file 1: Table S3).

Leucine can be used as both an electron donor and an acceptor in the Stickland reactions [37]. The *hadAIBC-acdB-etfBA* operon, which is involved in reduction of l-leucine to isocaproate, is preceded by a putative σ^54^ promoter and a candidate UAS site of LeuR in the genome of *C. difficile* (Fig. 6a, c). This suggests that reduction of leucine may be controlled by σ^54^ and LeuR in *C. difficile*. In addition, the *ord-ortBA-oraSEF-orr-nhaC*, which is involved in oxidation of ornithine to acetate, alanine, and ammonia, is predicted to be regulated by σ^54^ and OrdR in five species including *C. difficile*, *Romboutsia* sp. Frifi, *G. ferrireducens*, *Clostridium aceticum*, and *C. scatologenes* (Fig. 6a).

Utilization of cysteine and methionine was predicted to be controlled by σ^54^ in several pathogenic *Clostridiales* species (Fig. 6a). Availability of cysteine and methionine strongly affects production of toxins in these species [38, 39]. Recent studies have shown that σ^54^ and CdsR mediate the cysteine-dependent repression of toxin production in *C. difficile* [40, 41]. We identified a putative σ^54^ promoter and UAS site of CsdR upstream of *cdsB* gene involved in cysteine catabolism in *C. difficile*, *C. botulinum*, *C. sporogenes*, and *C. scatologenes* (Fig. 6a; Table 1). Moreover, the *mdeA-metT* operon, which is involved in transport and catabolism of methionine, is predicted to be regulated by σ^54^ and MdeR in *C. botulinum*, *C. tetani*, and 9 other *Clostridiales* species (Fig. 6a; Table 1).

### Regulation of butyrate and alcohols synthesis

The reconstructed σ^54^ regulons contain the genes associated with butyrate and alcohols synthesis in *Clostridiales* species. The *crt-hbd-thl-maoC-bcd-etfAB* operon, which is able to convert acetyl coenzyme A (acetyl-CoA) to butyryl-CoA, is preceded by a putative σ^54^ promoter and multiple UAS sites of CrbR in the genomes of *C. beijerinckii*, *C. carboxidivorans*, *C. botulinum*, and five other species (Fig. 6c**;** Additional file 1: Table S3). We predicted that the expression of this operon likely depends on the co-regulation of the CrbR and σ^54^ in these *Clostridiales* species, however the signal molecular remains unknown [42]. Candidate σ^54^ promoter was also identified in the upstream region of *adhA* and *adhA2* genes encoding alcohol dehydrogenases, *butA* encoding 2,3-butanediol dehydrogenase, and *sadh* encoding a secondary alcohol dehydrogenase [42–44] (Table 1). These genes constitute the most conserved part of the σ^54^ regulons in *Clostridiales* species. The corresponding EBPs are AdhR, BldR, and SadR, respectively (Fig. 6b). The *aor* gene encoding aldehyde oxidoreductase, which catalyzes the reduction of acetate to acetaldehyde, was predicted to be regulated by σ^54^ and AorR in *C. ljungdahlii*, *C. carboxidivorans*, *C. autoethanogenum*, and six other species. This gene has been shown to play an important role in ethanol production from syngas in *C. autoethanogenum* [45].

### Comparison of σ^54^ regulons between different *Clostridiales* species

**Fig. 7:**
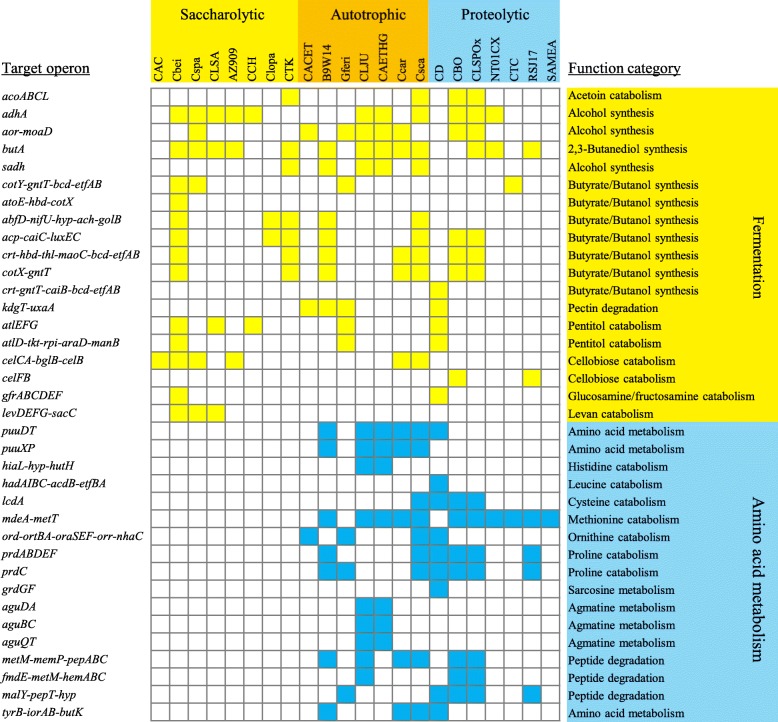
Distribution of predicted target operons of σ^54^ in *Clostridiales* species. The abbreviations of *Clostridiales* species are described in Fig. 5**.** The putative σ^54^-dependent genes involved in fermentation or amino acid metabolism are marked by yellow or blue square respectively

Clostridia are often differentiated by performing a saccharolytic or a proteolytic metabolism, although some proteolytic species can also grow on sugars [46]. Moreover, some saccharolytic species are able to perform autotrophic metabolism by using CO_2_/H_2_ gas mixture or CO as substrate [47]. We compared the reconstructed σ^54^ regulons between different *Clostridiales* species. In saccharolytic species such as *C. beijerinckii*, *C. butyricum*, and *C. saccharoperbutylacetonicum*, the σ^54^ regulons control sugar catabolism and fermentation, particularly butyrate and alcohols synthesis (Fig. 7). In proteolytic species such as *C. difficile*, *C. botulinum*, and *C. sporogenes*, the σ^54^ regulons contain not only the genes involved in amino acid catabolism (particularly in the Stickland reactions) but also the genes for sugar catabolism and fermentation (Fig. 7). Thus, the σ^54^ is likely closely linked to the central metabolism in different *Clostridiales* species. The size of the σ^54^ regulons is relatively large in the acetogenic species that are capable of autotrophic metabolism, including *C. ljungdahlii*, *C. carboxidivorans*, and *C. autoethanogenum*. Interestingly, for these species, the σ^54^ regulons control not only sugar catabolism and fermentation but also amino acid metabolism (Fig. 7). Previous studies have shown that the amino acid metabolism may provide reducing power and energy for autotrophic growth of *C. autoethanogenum* [48].

### Experimental validation of σ^54^ binding to predicted DNA targets

**Fig. 8:**
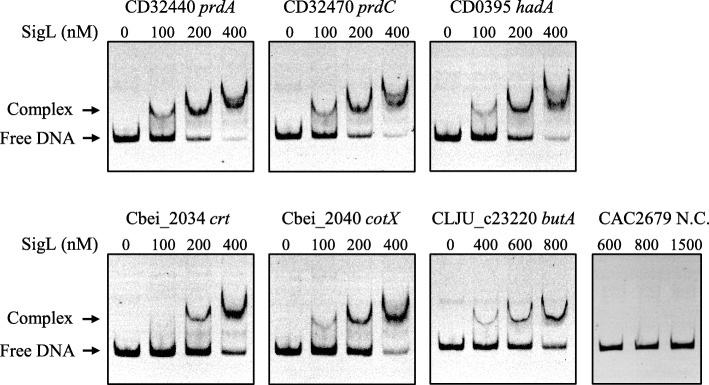
Experimental validation of the σ^54^ regulons in *Clostridiales* species. The EMSAs were performed with the purified SigL (σ^54^) protein from *C. beijerinckii* and DNA fragments containing the candidate −24 and − 12 regions upstream of predicted target genes in *Clostridiales* species. As a negative control (N.C.), the promoter region of CAC2679 gene in *C. acetobutylicum* was used, which lacks putative −12 and − 24 elements

Electrophoretic mobility shift assays (EMSAs) were performed with the recombinant SigL (σ^54^) protein from *C*. *beijerinckii* to validate the predicted clostridial σ^54^ regulons. The SigL (σ^54^) protein is well conserved in the analyzed clostridia. We tested six DNA fragments from the upstream region of *C. difficile prdC*, *prdABDE-prdE2-prdF*, *hadAIBC-acdB-etfBA*; *C. beijerinckii crt-bcd-etfAB-moaC*, *cotX-gntT*; and *C. ljungdahlii butA*. These DNA fragments contain the predicted σ^54^ promoter elements. Upon the incubation of SigL protein with each promoter fragment, a shifted band was observed, and its intensity was σ^54^ concentration-dependent increased (Fig. 8). In contrast, the DNA fragment that lacks putative σ^54^ promoter elements was not shifted even at 1500 nM SigL protein (Fig. 8). These results confirm that SigL (σ^54^) binds specifically to the promoter regions of the predicted σ^54^ regulon members involved in the Stickland reactions and butyrate and alcohols synthesis in *Clostridiales* species.

## Discussion

In this study, we performed comparative genomic reconstruction of transcriptional regulons of σ^54^ and 263 EBPs in 57 species from the *Clostridiales* order. These EBPs constitute 39 distinct groups. The sizes and gene contents of reconstructed σ^54^ regulons varied significantly among *Clostridiales* species. Based on the gene contents of the reconstructed regulons, the σ^54^ was predicted to control the central metabolism in diverse *Clostridiales* species. The predicted σ^54^ binding sites in the genomes of *Clostridiales* spp. were experimentally validated.

The reconstructed σ^54^ regulons contain the genes involved in fermentation and amino acid catabolism, particularly in the Stickland reactions and butyrate and alcohols synthesis. To the best of our knowledge, this is the first report about direct regulation of the Stickland reactions and butyrate and alcohols synthesis by σ^54^ and the respective EBPs. Thus, the σ^54^ was predicted to control the ethanol and butanol production in solvent-producing clostridia including *C. beijerinckii*, *C. saccharobutylicum*, and *C. saccharoperbutylacetonicum*. In pathogenic clostridia including *C. difficile*, *C. tetani*, and *C. botulinum*, the σ^54^ was proposed to regulate the amino acid catabolism, especially the Stickland reaction, which strongly influences the production of toxins [33, 34]. Thus, the σ^54^ is probably strongly linked to the virulence of these pathogenic species. For the acetogenic species including *C. ljungdahlii*, *C. carboxidivorans*, and *C. autoethanogenum* that can fix CO_2_ or CO [47], the σ^54^ may play an important role in regulation of both heterotrophic and autotrophic metabolism. Although the recent two studies in *C. beijerinckii* have obtained some similar results about the σ^54^ function [49, 50], our systematic analysis of the regulatory network of σ^54^ yielded more complete and comprehensive regulons of σ^54^ and EBPs, covering all completely sequenced *Clostridiales* genomes.

The majority of the EBPs in *Clostridiales* are OCSs possessing a regulatory domain that directly recognize signal molecules and modulates the activity of the EBPs. A variety of domains were present in the regulatory region of the EBPs in *Clostridiales*. The most frequently found domain is the PAS domain, which can sense oxygen, light, redox potential, and energy status through binding various cofactors and ligands [25, 26]. The PAS domain is usually present in two copies or adjacent to other domains such as the GAF domain that can also bind diverse small-molecule metabolites [51, 52]. These regulatory domains could allow the EBPs to sense various signals of intracellular environment such as redox and energy status. Even one EBP may respond to multiple input signals. Thus, the σ^54^-dependent transcription may enable a rapid regulation of the central metabolism in response to changes in various environmental conditions.

## Conclusions

In this study, we comprehensively characterized the σ^54^-dependent regulons in 57 *Clostridiales* species. In the analyzed genomes, we identified σ^54^ associated activators and their DNA-binding sites, as well as σ^54^-recognised promoters, and σ^54^-controlled genes and operons. In particular, we inferred σ^54^-dependent genes that are unknown before, including those involved in the Stickland reactions and butyrate and alcohols synthesis. Our results showed that the gene contexts and sizes of σ^54^-dependent regulons among *Clostridiales* species reveal significant difference. It is proposed that the σ^54^ controls butyrate and alcohols synthesis in solvent-producing species, regulates autotrophic metabolism in acetogenic species, and affects the toxin production in pathogenic species.

## Methods

### Identification of σ^54^ and enhancer binding proteins (EBPs)

Genomes analyzed in this study were download from GenBank [53], and were listed in the Additional file 1: Table S1. σ^54^ (SigL) orthologs were identified by similarity search using SigL from *Bacillus subtilis*. EBPs were identified based on homology to NtrC from *E. coli* and AcoR from *B. subtilis* using BLAST with an *E*-value threshold 1.0E-5. The presence of the characteristic amino acid motif GAFTGA was checked, which is required for the interaction between EBP and σ^54^ [54]. The MAFFT program [55] was used for protein sequence alignments. Conserved functional domains were identified using the HHpred tool [56] and Pfam [57]. Transmembrane regions were identified using the TMHMM server [58] and TMMOD [59]. Phylogenetic trees were constructed using the maximum-likelihood method implemented in MEGA [60], with calculation of bootstraps from 1000 replicates. The MicrobesOnline database [61] and GenomeExplorer software [62] were used for cross-genomic comparison of genomic contexts for EBPs. Spearman correlation test was applied to assess the association of the EBPs number with the genome size.

### Identification of σ^54^ promoters

For identification of σ^54^ binding sequences with conserved elements located at − 12 and − 24 positions, the 85 known promoters [30] were utilized to formulate the σ^54^ promoter sequence motif using the SignalX [62]. The motif was used to scan the genomes by the RegPredict [63] and GenomeExplorer [62] tools. The score threshold was defined as the lowest score observed in the training set.

### Reconstruction of regulons of EBPs

Transcriptional regulons of EBPs were reconstructed using an established comparative genomics method based on identification of candidate regulator-binding sites in closely related prokaryotic genomes [64]. For identification of the conserved UAS motif for EBPs, we constructed the training sets of potentially regulated operons that are co-localized with σ^54^ promoters and EBP-encoding genes on the chromosome. For each group of EBP orthologs, a separate training gene set was used. The upstream noncoding sequences of potentially regulated operons were extracted, and an iterative motif detection algorithm implemented in the RegPredict was used to identify the UAS motif. A positional weight matrix was constructed for the identified motif and used to search the upstream regions of coding genes (from − 400 to + 50 bp with respect to the translation start) for candidate UAS sites in the genomes using the RegPredict [63] and GenomeExplorer [62] tools. Scores of candidate UAS sites were calculated as the sum of positional nucleotide weights. The score threshold was defined as the lowest score observed in the training set. Genes with candidate upstream UAS sites that are high scored and/or conserved in two or more genomes were included in the regulon of the respective EBP. The UAS motifs were visualized as sequence logos using WebLogo [65].

Functional annotations of the reconstructed regulon members were based on the literature and MicrobesOnline [61]. Known functional assignment for a particular gene was expanded to its orthologous genes. For prediction of gene function, both the comparative genomics and context-based methods were used [64] .

### Protein overexpression and purification

The *sigL* gene was PCR amplified from *C. beijerinckii* NCIMB 8052 genomic DNA using the primers shown in Additional file 1: Table S4. The PCR fragment was ligated into the expression vector pET28a. The resulting plasmid pET28a-*sigL* was used to produce SigL protein with an N-terminal hexahistidine tag. *E. coli* BL21Rosetta(DE3) (Novagen) was transformed with expression plasmid. Protein overexpression and purification were performed as described previously [21].

### Electrophoretic mobility shift assay

The 200-bp DNA fragments in the promoter region of *crt* or *cotX* gene from *C. beijerinckii* genome and of *butA* gene from *C. ljungdahlii* genome were PCR amplified using the primers shown in Additional file 1: Table S4. The DNA fragments containing the putative promoter elements upstream of *prdA*, *prdC* and *hadA* genes from *C. difficile* were chemically synthesized by Genscript. Both forward and reverse primers were Cy5 fluorescence labeled at the 5′-end (Sangong, China). Mobility shift assays were performed as described previously [21].

## Additional file

Additional file 1: **Table S1.** List of *Clostridiales* species containing σ^54^ (SigL) and EBPs. **Table S2.** Identified EBPs in *Clostridiales*. **Table S3.** Reconstructed regulons of EBPs in *Clostridiales*. **Table S4.** Primers used in this study. (PDF 1577 kb)

## Data Availability

All data analyzed in this study were obtained from the NCBI (https://www.ncbi.nlm.nih.gov/) and MicrobesOnline [61]. The data sets supporting the results of this article are included within the article and its additional files.

## Abbreviations

AAA^+^: ATPase associated with cellular activities domain
DBD: DNA binding domain
EBPs: Enhancer binding proteins
GAF: Cyclic GMP-specific phosphodiesterases, adenylyl cyclases and FhlA
PAS: Per-Arnt-Sim domain
PRD: Phosphoenolpyruvate-dependent phosphotransferase system regulation domain
RR: Response regulator domain
UAS: Upstream activator sequences
