# Supplementary material for: Genomic reconstruction of σ54 regulons in Clostridiales
Source: BMC Genomics. 2019 Jul 9;20:565. doi: 10.1186/s12864-019-5918-4 (PMC6615313; doi:10.1186/s12864-019-5918-4)
Supplement: Supplementary file 1 — Table S1. List of Clostridiales species containing σ54 (SigL) and EBPs. Table S2. Identified EBPs in Clostridiales. Table S3. Reconstructed regulons of EBPs in Clostridiales. Table S4. Primers used in this study. (PDF 1577 kb) [file 12864_2019_5918_MOESM1_ESM.pdf]

Supplementary Information

Genomic Reconstruction of σ^{54} Regulons in *Clostridiales*

Xiaoqun Nie, Wenyue Dong, Chen Yang

Table S1 List of *Clostridiales* species containing σ^{54} (SigL) and EBPs.

Table S2 Identified EBPs in *Clostridiales*.

Table S3 Reconstructed regulons of EBPs in *Clostridiales*.

Table S4 Primers used in this study.

Table S1. List of *Clostridiales* species containing σ^{54} (SigL) and EBPs

Genus	Organism	Genome size (Mb)	Locus tag of σ^{54} (SigL)	Number of EBPs
Acetobacterium	*Acetobacterium* sp. KB-1	3.99	DOZ58_06690	5
Acetobacterium	*Acetobacterium woodii* DSM 1030	4	Awo_c24550	6
Alkaliphilus	*Alkaliphilus metalliredigens* QYMF	4.93	Amet_3582	8
Alkaliphilus	*Alkaliphilus oremlandii* OhILAs	3.12	Clos_0967	8
Carboxydocella	*Carboxydocella thermautotrophica* strain 019	2.68	CFE_0544	9
Clostridioides	*Clostridioides difficile* 630	4.3	CD3176	23
Clostridium	*Clostridium aceticum* strain DSM 1496	4.21	CACET_RS06065	8
Clostridium	*Clostridium acetobutylicum* ATCC 824	4.13	CA_C0707	3
Clostridium	*Clostridium argentinense* strain 89G	4.8	RSJ17_15205	9
Clostridium	*Clostridium autoethanogenum* DSM 10061	4.35	CAETHG_1762	12
Clostridium	*Clostridium baratii* str. Sullivan	3.34	U729_342	6
Clostridium	*Clostridium beijerinckii* NCIMB 8052	6	Cbei_0595	19
Clostridium	*Clostridium botulinum* A str. ATCC 3502	3.9	CBO0224	12
Clostridium	*Clostridium butyricum* strain TOA	4.6	AZ909_02870	4
Clostridium	*Clostridium carboxidivorans* P7	5.75	Ccar_RS22190	11
Clostridium	*Clostridium chauvoei* JF4335	2.89	CCH01_21620	2
Clostridium	*Clostridium cochlearium* strain NCTC13027	2.44	SAMEA4530647_00243	3
Clostridium	*Clostridium drakei* strain SL1	5.7	B9W14_00390	17
Clostridium	*Clostridium estertheticum* DSM 8809	4.79	A7L45_19510	6
Clostridium	*Clostridium formicaceticum* strain ATCC 27076	4.59	BJL90_19735	9
Clostridium	*Clostridium kluyveri* DSM 555	4.02	CKL_3013	4
Clostridium	*Clostridium ljungdahlii* DSM 13528	4.63	CLJU_c39170	19
Clostridium	*Clostridium novyi* NT	2.55	NT01CX_1402	4
Clostridium	*Clostridium pasteurianum* BC1	5.04	Clopa_3638	6
Clostridium	*Clostridium perfringens* ATCC 13124	3.26	CPF_1513	1
Clostridium	*Clostridium saccharobutylicum* DSM 13864	5.11	CLSA_RS19450	5
Clostridium	*Clostridium saccharoperbutylacetonicum* N1-4(HMT)	6.67	Cspa_c51140	14
Clostridium	*Clostridium scatologenes* ATCC 25775	5.75	Csca_RS18330	30
Clostridium	*Clostridium sporogenes*strain DSM 795	4.14	CLSPOx_01280	13
Clostridium	*Clostridium taeniosporum* strain 1/k	3.5	BGI42_12055	6
Clostridium	*Clostridium tetani* E88	2.87	CTC00376	6
Clostridium	*Clostridium tyrobutyricum* strain KCTC 5387	3.13	CTK_RS01220	11
Dehalobacter	*Dehalobacter restrictus* DSM 9455	2.94	DEHRE_12030	2
Dehalobacter	*Dehalobacter* sp. CF	3.09	DCF50_p1650	1
Dehalobacter	*Dehalobacter* sp. DCA	3.07	DHBDCA_p1641	1
Dehalobacterium	*Dehalobacterium formicoaceticum* strain DMC	3.77	CEQ75_RS01830	11
Desulfitobacterium	*Desulfitobacterium hafniense* DCB-2	5.28	Dhaf_4719	17
Desulfosporosinus	*Desulfosporosinus orientis* DSM 765	5.86	Desor_5473	35
Desulfotomaculum	*Desulfotomaculum ferrireducens* strain GSS09	3.24	B0537_15150	6
Desulfotomaculum	*Desulfotomaculum reducens* MI-1	3.61	Dred_2992	10
Desulfotomaculum	*Desulfotomaculum ruminis* DSM 2154	3.97	Desru_3600	9
Eubacterium	*Eubacterium limosum* KIST612	4.28	ELI_0659	6
Eubacterium	*Eubacterium sulci* ATCC 35585	1.74	ADJ67_04095	3
Flavonifractor	*Flavonifractor plautii* strain YL31	3.82	A4U99_14675	9
Geosporobacter	*Geosporobacter ferrireducens* strain IRF9	5.82	Gferi_24440	23
Heliobacterium	*Heliobacterium modesticaldum* Ice1	3.08	HM1_1310	2
Intestinimonas	*Intestinimonas butyriciproducens* strain af211	3.38	IB211_02653c	7
Lachnoclostridium	*[Clostridium] bolteae* strain ATCC BAA-613	6.61	CGC65_27965	6
Lachnoclostridium	*Lachnoclostridium* sp. YL32	7.22	A4V08_19805	5
Oscillibacter	*Oscillibacter valericigenes* Sjm18-20	4.47	OBV_07640	4
Pelotomaculum	*Pelotomaculum thermopropionicum* SI	3.03	PTH_2724	7
Romboutsia	*Romboutsia ilealis* strain CRIB	2.59	CRIB_185	1
Romboutsia	*Romboutsia* sp. Frifi	3.04	FRIFI_0179	4
Symbiobacterium	*Symbiobacterium thermophilum* IAM 14863	3.57	STH239	6
Syntrophomonas	*Syntrophomonas wolfei* str. Goettingen	2.94	Swol_0265	6
Syntrophothermus	*Syntrophothermus lipocalidus* DSM 12680	2.41	Slip_1854	6
Thermincola	*Thermincola potens* JR	3.16	TherJR_2713	4

Table S2 Identified EBPs in *Clostridiales*

Locus tag of EBP	Organism	OCS	"GAFTGA" motif	N-terminal regulatory domain	DBD binding
Amet_0225	*Alkaliphilus metalliredigens* QYMF	Y	GAFTGA	PrpR_N-PAS	HTH_8
Amet_0663	*Alkaliphilus metalliredigens* QYMF	Y	GAFTGA	CBS-CBS-PAS	HTH_8
Amet_2060	*Alkaliphilus metalliredigens* QYMF	Y	GAFTGA	+	HTH_8
Amet_4197	*Alkaliphilus metalliredigens* QYMF	Y	GAYTGA	PRD-EIIA-PRD	TrmB
Amet_4245	*Alkaliphilus metalliredigens* QYMF	Y	GSFTGA	PAS	HTH_8
Amet_4248	*Alkaliphilus metalliredigens* QYMF	Y	GAFTGA	PAS	HTH_8
Amet_4644	*Alkaliphilus metalliredigens* QYMF	Y	GAFTGA	GAF-PAS	HTH_8
Amet_4666	*Alkaliphilus metalliredigens* QYMF	Y	GAFTGA	GAF-PAS	HTH_8
Clos_0026	*Alkaliphilus oremlandii* OhILAs	Y	GAFTGA	CBS-CBS-PAS	HTH_8
Clos_0048	*Alkaliphilus oremlandii* OhILAs	Y	GSFTGA	PrpR_N-PAS	HTH_8
Clos_0069	*Alkaliphilus oremlandii* OhILAs	Y	GAFTGA	PAS	+
Clos_0085	*Alkaliphilus oremlandii* OhILAs	Y	GAFTGA	PAS	HTH_8
Clos_0171	*Alkaliphilus oremlandii* OhILAs	Y	GSFTGA	PAS	HTH_8
Clos_1806	*Alkaliphilus oremlandii* OhILAs	Y	GAFTGA	PAS	HTH_8
Clos_2445	*Alkaliphilus oremlandii* OhILAs	Y	GAFTGA	PAS	HTH_8
Clos_2825	*Alkaliphilus oremlandii* OhILAs	N	GAFTGA	RR	HTH_8
CACET_RS01495	*Clostridium aceticum* strain DSM 1496	N	GAFTGA	RR	HTH_8
CACET_RS01850	*Clostridium aceticum* strain DSM 1496	N	GAFSGA	RR	HTH_8
CACET_RS02445	*Clostridium aceticum* strain DSM 1496	Y	GAFTGA	GAF-PAS	HTH_8
CACET_RS08525	*Clostridium aceticum* strain DSM 1496	Y	GAFTGA	PAS	HTH_8
CACET_RS14445	*Clostridium aceticum* strain DSM 1496	Y	GAFTGA	GAF	HTH_8
CACET_RS18225	*Clostridium aceticum* strain DSM 1496	Y	GAFTGA	GAF-PAS	HTH_8
CACET_RS18720	*Clostridium aceticum* strain DSM 1496	Y	GAFTGA	PrpR_N-PAS	HTH_8
CACET_RS18820	*Clostridium aceticum* strain DSM 1496	Y	GAFTGS	PAS	HTH_8
CA_C0382	*Clostridium acetobutylicum* ATCC 824	Y	GTYTGA	PRD-EIIA-PRD	+
CA_C0459	*Clostridium acetobutylicum* ATCC 824	Y	GSFTGA	PrpR_N-PAS	HTH_8
CA_C3088	*Clostridium acetobutylicum* ATCC 824	Y	GAFTGA	PTS_HPr-PAS-PAS	+
RSJ17_01135	*Clostridium argentinense* strain 89G	N	GAFTGA	RR	HTH_8
RSJ17_05060	*Clostridium argentinense* strain 89G	Y	GAFTGA	CBS-CBS-PAS	HTH_8
RSJ17_06280	*Clostridium argentinense* strain 89G	Y	GAFSGA	GAF-PAS	HTH_8
RSJ17_06895	*Clostridium argentinense* strain 89G	Y	GSFTGA	PAS	HTH_8
RSJ17_08670	*Clostridium argentinense* strain 89G	Y	GSFTGA	PAS	HTH_8
RSJ17_13385	*Clostridium argentinense* strain 89G	Y	GAYTGA	PRD-EIIA-PRD	TrmB
RSJ17_16220	*Clostridium argentinense* strain 89G	Y	GVFTAG	PAS	HTH_8
RSJ17_16990	*Clostridium argentinense* strain 89G	Y	GAFTGA	GAF-PAS	HTH_8
RSJ17_17665	*Clostridium argentinense* strain 89G	Y	GAFTGA	+	HTH_12
CAETHG_0101	*Clostridium autoethanogenum* DSM 10061	Y	GAFTGA	0	HTH_8
CAETHG_0105	*Clostridium autoethanogenum* DSM 10061	Y	GAFTGA	GAF-PAS	HTH_8
CAETHG_0386	*Clostridium autoethanogenum* DSM 10061	Y	GAFTGA	GAF-PAS	HTH_8
CAETHG_0472	*Clostridium autoethanogenum* DSM 10061	Y	GAFTGA	GAF-PAS	HTH_8
CAETHG_0552	*Clostridium autoethanogenum* DSM 10061	Y	GSFTGA	GAF-PAS	HTH_8
CAETHG_0554	*Clostridium autoethanogenum* DSM 10061	Y	GSFTGA	GAF-PAS	HTH_8
CAETHG_1186	*Clostridium autoethanogenum* DSM 10061	Y	GAFTGS	PAS	HTH_8
CAETHG_1538	*Clostridium autoethanogenum* DSM 10061	Y	GSFTGA	PAS-PAS	HTH_8
CAETHG_1556	*Clostridium autoethanogenum* DSM 10061	Y	GAFTGA	PAS	+
CAETHG_1910	*Clostridium autoethanogenum* DSM 10061	Y	GAFTGA	GAF-PAS	+
CAETHG_2076	*Clostridium autoethanogenum* DSM 10061	Y	GAFTGA	0	HTH_8
CAETHG_2800	*Clostridium autoethanogenum* DSM 10061	Y	GAFTGA	PAS	HTH_8
U729_1576	*Clostridium baratii* str. Sullivan	Y	GAYTGA	PRD-EIIA-PRD	TrmB
U729_2501	*Clostridium baratii* str. Sullivan	Y	GAFTGA	PRD-EIIA-PRD	HTH_24
U729_2760	*Clostridium baratii* str. Sullivan	Y	GAFTGA	+	HTH_8
U729_324	*Clostridium baratii* str. Sullivan	Y	GAFTGA	GAF-PAS	HTH_8
U729_560	*Clostridium baratii* str. Sullivan	Y	GTFTGA	PRD	TrmB
U729_973	*Clostridium baratii* str. Sullivan	Y	GAFTGA	PRD-EIIA-PRD	+
Cbei_0219	*Clostridium beijerinckii* NCIMB 8052	Y	GAYTGA	PRD-EIIA-PRD	+

Cbei_0540	*Clostridium beijerinckii* NCIMB 8052	Y	GAFTGA	PRD-EIIA-PRD	+
Cbei_0953	*Clostridium beijerinckii* NCIMB 8052	Y	GAYTGA	PRD-EIIA-PRD	+
Cbei_0954	*Clostridium beijerinckii* NCIMB 8052	Y	GAFTGA	PRD-EIIA-PRD	+
Cbei_0962	*Clostridium beijerinckii* NCIMB 8052	Y	GAYTGA	PRD-EIIA-PRD	+
Cbei_1463	*Clostridium beijerinckii* NCIMB 8052	Y	GAFTGA	GAF-PAS	HTH_8
Cbei_2039	*Clostridium beijerinckii* NCIMB 8052	Y	GAFTGA	PAS-PAS	HTH_8
Cbei_2099	*Clostridium beijerinckii* NCIMB 8052	Y	GAFTGA	CBS-PAS	HTH_8
Cbei_2147	*Clostridium beijerinckii* NCIMB 8052	Y	GAFTGA	PTS_HPr-PAS-PAS	+
Cbei_2180	*Clostridium beijerinckii* NCIMB 8052	Y	GTFTGG	GAF-PAS	HTH_8
Cbei_2497	*Clostridium beijerinckii* NCIMB 8052	Y	GAFTGA	PRD-EIIA-PRD	+
Cbei_3352	*Clostridium beijerinckii* NCIMB 8052	Y	GAFTGA	PAS-PAS	HTH_8
Cbei_3816	*Clostridium beijerinckii* NCIMB 8052	N	GAFTSA	RR	HTH_8
Cbei_3875	*Clostridium beijerinckii* NCIMB 8052	Y	GAFTGA	PRD-EIIA-PRD	+
Cbei_4539	*Clostridium beijerinckii* NCIMB 8052	Y	GAFTGA	PRD-EIIA-PRD	+
Cbei_4555	*Clostridium beijerinckii* NCIMB 8052	Y	GAFTGA	PRD-EIIA-PRD	+
Cbei_4641	*Clostridium beijerinckii* NCIMB 8052	Y	GAYTGA	PRD-EIIA-PRD	+
Cbei_4686	*Clostridium beijerinckii* NCIMB 8052	Y	GAYTGA	PRD-EIIA-PRD	HTH_11
Cbei_4915	*Clostridium beijerinckii* NCIMB 8052	Y	GAFTGA	PRD-EIIA-PRD	+
CBO0028	*Clostridium botulinum* A str. ATCC 3502	Y	GSFTGA	ACT-ACT-PAS	+
CBO0594	*Clostridium botulinum* A str. ATCC 3502	Y	GAFTGA	PAS	HTH_8
CBO1628	*Clostridium botulinum* A str. ATCC 3502	Y	SAFTGA	GAF-PAS	HTH_8
CBO1642	*Clostridium botulinum* A str. ATCC 3502	Y	GAFTGA	GAF-PAS	HTH_8
CBO1937	*Clostridium botulinum* A str. ATCC 3502	Y	GAFTGA	GAF-PAS	HTH_8
CBO1959	*Clostridium botulinum* A str. ATCC 3502	Y	GAFTGA	PrpR_N-PAS	+
CBO2009	*Clostridium botulinum* A str. ATCC 3502	Y	GAFSGA	GAF-PAS	+
CBO2490	*Clostridium botulinum* A str. ATCC 3502	Y	GTFTGS	CBS-CBS-PAS	HTH_8
CBO2889	*Clostridium botulinum* A str. ATCC 3502	Y	GAFSGA	GAF-PAS	HTH_8
CBO3196	*Clostridium botulinum* A str. ATCC 3502	Y	GAFTGA	PAS-PAS	HTH_8
CBO3358	*Clostridium botulinum* A str. ATCC 3502	Y	GAYTGA	PRD-EIIA-PRD	+
CBO3439	*Clostridium botulinum* A str. ATCC 3502	Y	GAFTGA	PTS_HPr-PAS-PAS	+
AZ909_01580	*Clostridium butyricum* strain TOA	Y	GAFTGA	PRD-EIIA-PRD	MarR_2
AZ909_04455	*Clostridium butyricum* strain TOA	Y	GAFTGA	GAF-PAS	HTH_8
AZ909_14335	*Clostridium butyricum* strain TOA	Y	GAYTGA	PRD-EIIA-PRD	+
AZ909_16155	*Clostridium butyricum* strain TOA	Y	GTFTGA	GAF-PAS	HTH_8
Ccar_RS00040	*Clostridium carboxidivorans* P7	Y	GSFTGA	PAS	NUMOD1
Ccar_RS01435	*Clostridium carboxidivorans* P7	Y	GAFTGA	PAS-PAS	HTH_8
Ccar_RS01625	*Clostridium carboxidivorans* P7	Y	GAFTGA	PAS	+
Ccar_RS04445	*Clostridium carboxidivorans* P7	Y	GAFTGA	GAF-PAS	HTH_8
Ccar_RS07070	*Clostridium carboxidivorans* P7	Y	GSFTGA	PAS	+
Ccar_RS07685	*Clostridium carboxidivorans* P7	Y	GAFTGA	PRD-EIIA-PRD	+
Ccar_RS12725	*Clostridium carboxidivorans* P7	Y	GAFTGA	0	HTH_8
Ccar_RS15630	*Clostridium carboxidivorans* P7	Y	GAFTGA	PrpR_N-PAS	HTH_8
Ccar_RS16040	*Clostridium carboxidivorans* P7	Y	GAFTGA	PAS	HTH_8
Ccar_RS17545	*Clostridium carboxidivorans* P7	Y	GAFTGA	GAF-PAS	+
Ccar_RS19565	*Clostridium carboxidivorans* P7	Y	GAFTGA	PTS_HPr-PAS-PAS	+
CCH01_21380	*Clostridium chauvoei* JF4335	Y	GTFTGG	GAF-PAS	HTH_8
CCH01_24820	*Clostridium chauvoei* JF4335	Y	GSFTGA	PAS	HTH_8
SAMEA4530647_00452	*Clostridium cochlearium* strain NCTC13027	Y	GAFTGA	GAF-PAS	HTH_8
SAMEA4530647_01995	*Clostridium cochlearium* strain NCTC13027	N	GAFTGA	RR	HTH_8
SAMEA4530647_02232	*Clostridium cochlearium* strain NCTC13027	Y	GSFTGA	ACT-PAS	HTH_8
B9W14_01155	*Clostridium drakei* strain SL1	Y	GAFTGA	PAS	HTH_8
B9W14_01575	*Clostridium drakei* strain SL1	Y	GAFTGA	PAS	HTH_8
B9W14_04855	*Clostridium drakei* strain SL1	Y	GSFTGA	ACT-PAS	HTH_8
B9W14_06530	*Clostridium drakei* strain SL1	Y	GAFTGA	PAS-PAS	HTH_8
B9W14_06665	*Clostridium drakei* strain SL1	Y	GAFTGA	GAF-PAS	+
B9W14_09360	*Clostridium drakei* strain SL1	Y	GAFTGA	GAF-PAS	HTH_8
B9W14_11630	*Clostridium drakei* strain SL1	Y	GSFTGA	PAS	HTH_12
B9W14_12220	*Clostridium drakei* strain SL1	Y	GAFTGA	PRD-EIIA-PRD	TrmB
B9W14_13255	*Clostridium drakei* strain SL1	Y	GAFTGA	PAS	HTH_8
B9W14_14690	*Clostridium drakei* strain SL1	Y	GAFTGA	PrpR_N-PAS	HTH_8

B9W14_19680	*Clostridium drakei* strain SL1	Y	GAFTGA	PrpR_N-PAS	HTH_8
B9W14_20055	*Clostridium drakei* strain SL1	Y	GAFTGA	PAS	HTH_8
B9W14_20635	*Clostridium drakei* strain SL1	Y	GAFTGA	PrpR_N-PAS	HTH_8
B9W14_21275	*Clostridium drakei* strain SL1	Y	GAFTGA	GAF-PAS	HTH_8
B9W14_22495	*Clostridium drakei* strain SL1	Y	GAFTGA	PAS	HTH_8
B9W14_23350	*Clostridium drakei* strain SL1	Y	GAFTGA	PTS_HPr-PAS-PAS	HTH_8
B9W14_25175	*Clostridium drakei* strain SL1	Y	GAFTGA	CBS-CBS-PAS	HTH_8
A7L45_02315	*Clostridium estertheticum* DSM 8809	Y	GAFTGA	PAS	HTH_8
A7L45_02395	*Clostridium estertheticum* DSM 8809	Y	GAFTGA	PAS-PAS	HTH_8
A7L45_04240	*Clostridium estertheticum* DSM 8809	Y	GAFTGA	GAF-PAS	HTH_8
A7L45_07235	*Clostridium estertheticum* DSM 8809	Y	GSFTGA	GAF-PAS	HTH_8
A7L45_08625	*Clostridium estertheticum* DSM 8809	Y	GAFTGA	PRD-EIIA	+
A7L45_11990	*Clostridium estertheticum* DSM 8809	Y	GAFTGA	CBS-PAS	HTH_8
BJL90_01845	*Clostridium formicaceticum* strain ATCC 27076	N	GAFTGA	RR	HTH_8
BJL90_04430	*Clostridium formicaceticum* strain ATCC 27076	Y	GAFTGA	GAF-PAS	HTH_8
BJL90_04660	*Clostridium formicaceticum* strain ATCC 27076	Y	GAFTGA	PrpR_N-PAS	HTH_8
BJL90_08085	*Clostridium formicaceticum* strain ATCC 27076	Y	GAFTGA	GAF-PAS	HTH_8
BJL90_08125	*Clostridium formicaceticum* strain ATCC 27076	Y	GAFTGA	PrpR_N-PAS	HTH_8
BJL90_08895	*Clostridium formicaceticum* strain ATCC 27076	Y	GAFTGA	GAF-PAS	HTH_8
BJL90_11410	*Clostridium formicaceticum* strain ATCC 27076	Y	GSFTGA	PAS	HTH_8
BJL90_15420	*Clostridium formicaceticum* strain ATCC 27076	Y	GAFTGA	PrpR_N-PAS	HTH_8
BJL90_18420	*Clostridium formicaceticum* strain ATCC 27076	Y	GAFTGA	0	HTH_8
CKL_0542	*Clostridium kluyveri* DSM 555	Y	GAFTGA	GAF-PAS	HTH_8
CKL_2272	*Clostridium kluyveri* DSM 555	Y	GSFTGA	GAF-PAS	HTH_8
CKL_3021	*Clostridium kluyveri* DSM 555	Y	GAFTGA	CBS-PAS	HTH_8
CKL_3438	*Clostridium kluyveri* DSM 555	Y	GAFTGA	GAF-PAS	HTH_8
CLJU_c07090	*Clostridium ljungdahlii* DSM 13528	Y	GAFTGA	PAS	HTH_8
CLJU_c20120	*Clostridium ljungdahlii* DSM 13528	Y	GAFTGA	PAS	HTH_8
CLJU_c20130	*Clostridium ljungdahlii* DSM 13528	Y	GAFTGA	GAF-PAS	HTH_8
CLJU_c20200	*Clostridium ljungdahlii* DSM 13528	Y	GAFTGA	0	HTH_8
CLJU_c20240	*Clostridium ljungdahlii* DSM 13528	Y	GAFTGA	GAF-PAS	HTH_8
CLJU_c22680	*Clostridium ljungdahlii* DSM 13528	Y	GAFTGA	PAS	HTH_8
CLJU_c23230	*Clostridium ljungdahlii* DSM 13528	Y	GAFTGA	GAF-PAS	HTH_8
CLJU_c23970	*Clostridium ljungdahlii* DSM 13528	Y	GAFTGA	GAF-PAS	HTH_8
CLJU_c24060	*Clostridium ljungdahlii* DSM 13528	Y	GAFTGA	GAF-PAS	HTH_8
CLJU_c24140	*Clostridium ljungdahlii* DSM 13528	Y	GAFTGA	GAF-PAS	HTH_8
CLJU_c24850	*Clostridium ljungdahlii* DSM 13528	Y	GSFTGA	GAF-PAS	HTH_8
CLJU_c24870	*Clostridium ljungdahlii* DSM 13528	Y	GSFTGA	GAF-PAS	HTH_8
CLJU_c32880	*Clostridium ljungdahlii* DSM 13528	Y	GAFTGS	PAS-PAS	HTH_8
CLJU_c36300	*Clostridium ljungdahlii* DSM 13528	Y	GSFTGA	0	HTH_8
CLJU_c36950	*Clostridium ljungdahlii* DSM 13528	Y	GAFTGA	PrpR_N-PAS	+
CLJU_c40410	*Clostridium ljungdahlii* DSM 13528	Y	GAFTGA	GAF-PAS	HTH_8
CLJU_c40670	*Clostridium ljungdahlii* DSM 13528	Y	GAFTGA	GAF-PAS	+
CLJU_c42510	*Clostridium ljungdahlii* DSM 13528	Y	GAFTGA	PAS	HTH_8
CLJU_c42520	*Clostridium ljungdahlii* DSM 13528	Y	GAFTGA	PAS	HTH_8
NT01CX_0150	*Clostridium novyi* NT	Y	GSFTGA	ACT-ACT-PAS	HTH_8
NT01CX_0345	*Clostridium novyi* NT	Y	GAFTGA	GAF-PAS	HTH_8
NT01CX_0397	*Clostridium novyi* NT	Y	GAFTGA	PAS	+
NT01CX_0469	*Clostridium novyi* NT	Y	GAFTGA	GAF-PAS	HTH_8
Clopa_0392	*Clostridium pasteurianum* BC1	Y	GAFTGA	GAF-PAS	HTH_8
Clopa_0711	*Clostridium pasteurianum* BC1	Y	GAFTGA	PRD-EIIA	+
Clopa_0816	*Clostridium pasteurianum* BC1	Y	GTFTGA	PrpR_N-PAS	HTH_8
Clopa_1482	*Clostridium pasteurianum* BC1	Y	GAFTGA	V4R-PAS	HTH_8
Clopa_4010	*Clostridium pasteurianum* BC1	Y	GAFTGA	PAS	HTH_8
Clopa_4465	*Clostridium pasteurianum* BC1	Y	GAFTGA	PTS_HPr-PAS-PAS	+
CPF_2667	*Clostridium perfringens* ATCC 13124	Y	GAFTGA	PTS_HPr-PAS-PAS	+
CLSA_RS07870	*Clostridium saccharobutylicum* DSM 13864	Y	GTFTGG	GAF-PAS	HTH_8
CLSA_RS08780	*Clostridium saccharobutylicum* DSM 13864	Y	GAFTGA	PRD-EIIA-PRD	+
CLSA_RS09985	*Clostridium saccharobutylicum* DSM 13864	Y	GAFTGA	PRD-EIIA-PRD	+
CLSA_RS19605	*Clostridium saccharobutylicum* DSM 13864	Y	GAFTGA	GAF-PAS	HTH_8

CLSA_RS21470	*Clostridium saccharobutylicum* DSM 13864	Y	GAFTGA	PRD-EIIA-PRD	+
Cspa_c06870	*Clostridium saccharoperbutylacetonicum* N1-4(HMT)	Y	GAFTGA	GAF-PAS	HTH_8
Cspa_c17380	*Clostridium saccharoperbutylacetonicum* N1-4(HMT)	Y	GAYTGA	PRD-EIIA-PRD	+
Cspa_c20840	*Clostridium saccharoperbutylacetonicum* N1-4(HMT)	Y	GAFTGA	PAS-PAS	HTH_8
Cspa_c21140	*Clostridium saccharoperbutylacetonicum* N1-4(HMT)	Y	GAFTGA	V4R	HTH_8
Cspa_c22060	*Clostridium saccharoperbutylacetonicum* N1-4(HMT)	Y	GAFTGA	GAF-PAS	HTH_8
Cspa_c22700	*Clostridium saccharoperbutylacetonicum* N1-4(HMT)	Y	GAFTGA	PAS	HTH_8
Cspa_c27820	*Clostridium saccharoperbutylacetonicum* N1-4(HMT)	Y	GTFTGA	GAF-PAS	HTH_8
Cspa_c29880	*Clostridium saccharoperbutylacetonicum* N1-4(HMT)	Y	GAFTGA	PAS	+
Cspa_c44160	*Clostridium saccharoperbutylacetonicum* N1-4(HMT)	Y	GAFTGA	PrpR_N-PAS	HTH_8
Cspa_c49170	*Clostridium saccharoperbutylacetonicum* N1-4(HMT)	Y	GAYTGA	PRD-EIIA-PRD	+
Cspa_c50010	*Clostridium saccharoperbutylacetonicum* N1-4(HMT)	Y	GAFTGA	PrpR_N-PAS	HTH_8
Cspa_c53690	*Clostridium saccharoperbutylacetonicum* N1-4(HMT)	Y	GAYTGA	PRD-EIIA-PRD	+
Cspa_c53790	*Clostridium saccharoperbutylacetonicum* N1-4(HMT)	Y	GAYTGA	PRD-EIIA-PRD	HTH_11
Cspa_c55920	*Clostridium saccharoperbutylacetonicum* N1-4(HMT)	Y	GAFTGA	PRD-EIIA-PRD	+
Csca_RS00355	*Clostridium scatologenes* ATCC 25775	Y	GSFTGA	GAF-PAS	HTH_8
Csca_RS00565	*Clostridium scatologenes* ATCC 25775	Y	GSFTGA	PAS	HTH_8
Csca_RS03615	*Clostridium scatologenes* ATCC 25775	Y	GAFTGA	PrpR_N-PAS	HTH_8
Csca_RS05135	*Clostridium scatologenes* ATCC 25775	Y	GAFTGA	PAS	HTH_8
Csca_RS05205	*Clostridium scatologenes* ATCC 25775	Y	GAFTGA	GAF-PAS	HTH_8
Csca_RS06260	*Clostridium scatologenes* ATCC 25775	Y	GAFTGA	PRD-EIIA-PRD	+
Csca_RS06870	*Clostridium scatologenes* ATCC 25775	Y	GAFTGA	0	HTH_8
Csca_RS07205	*Clostridium scatologenes* ATCC 25775	Y	GSFTGA	0	+
Csca_RS09730	*Clostridium scatologenes* ATCC 25775	Y	GAFTGA	GAF-PAS	HTH_8
Csca_RS10080	*Clostridium scatologenes* ATCC 25775	Y	GAYTGS	PAS	+
Csca_RS10800	*Clostridium scatologenes* ATCC 25775	Y	GSFTGA	GAF-PAS	HTH_8
Csca_RS11550	*Clostridium scatologenes* ATCC 25775	Y	GAFTGA	GAF-PAS	+
Csca_RS11910	*Clostridium scatologenes* ATCC 25775	Y	GSYTGA	PAS	HTH_8
Csca_RS12425	*Clostridium scatologenes* ATCC 25775	Y	GAFTGA	PAS	+
Csca_RS12550	*Clostridium scatologenes* ATCC 25775	Y	GAFTGA	PAS-PAS	HTH_8
Csca_RS14075	*Clostridium scatologenes* ATCC 25775	Y	GSFTGA	ACT-PAS	HTH_8
Csca_RS14125	*Clostridium scatologenes* ATCC 25775	Y	GAFTGA	GAF-PAS	HTH_8
Csca_RS15485	*Clostridium scatologenes* ATCC 25775	Y	GAFTGA	GAF-PAS	HTH_8
Csca_RS17250	*Clostridium scatologenes* ATCC 25775	Y	GSFTGA	PAS	+
Csca_RS17305	*Clostridium scatologenes* ATCC 25775	Y	GAFTGA	PAS	HTH_8
Csca_RS17650	*Clostridium scatologenes* ATCC 25775	Y	GAFTGA	PAS	HTH_8
Csca_RS19145	*Clostridium scatologenes* ATCC 25775	Y	GAFTGA	CBS-CBS-PAS	HTH_8
Csca_RS21060	*Clostridium scatologenes* ATCC 25775	Y	GAFTGA	PTS_HPr-PAS-PAS	+
Csca_RS21860	*Clostridium scatologenes* ATCC 25775	Y	GAFTGA	PAS	+
Csca_RS21990	*Clostridium scatologenes* ATCC 25775	Y	GAFTGA	PAS	HTH_8
Csca_RS22215	*Clostridium scatologenes* ATCC 25775	N	GAFTGA	RR	HTH_8
Csca_RS23000	*Clostridium scatologenes* ATCC 25775	Y	GAFTGA	GAF-PAS	+
Csca_RS23790	*Clostridium scatologenes* ATCC 25775	Y	GAFTGA	PrpR_N-PAS	HTH_8
Csca_RS24105	*Clostridium scatologenes* ATCC 25775	Y	GAFTGA	PAS	HTH_8
Csca_RS24460	*Clostridium scatologenes* ATCC 25775	Y	GAFTGA	PrpR_N-PAS	HTH_8
CLSPOx_00150	*Clostridium sporogenes* strain DSM 795	Y	GSFTGA	ACT-PAS	HTH_38
CLSPOx_03155	*Clostridium sporogenes* strain DSM 795	Y	GAFTGA	PAS	HTH_8
CLSPOx_08485	*Clostridium sporogenes* strain DSM 795	Y	SAFTGA	PAS-PAS	HTH_8
CLSPOx_08650	*Clostridium sporogenes* strain DSM 795	Y	GAFTGA	GAF-PAS	HTH_8
CLSPOx_08670	*Clostridium sporogenes* strain DSM 795	Y	GAFTGA	GAF-PAS	HTH_8
CLSPOx_09730	*Clostridium sporogenes* strain DSM 795	Y	GAFTGA	GAF-PAS	HTH_8
CLSPOx_09870	*Clostridium sporogenes* strain DSM 795	Y	GAFTGA	+	+
CLSPOx_10150	*Clostridium sporogenes* strain DSM 795	Y	GAFSGA	GAF-PAS	+
CLSPOx_12930	*Clostridium sporogenes* strain DSM 795	Y	GTFTGS	CBS-CBS-PAS	HTH_8
CLSPOx_14815	*Clostridium sporogenes* strain DSM 795	Y	GAFSGA	GAF-PAS	HTH_8
CLSPOx_16515	*Clostridium sporogenes* strain DSM 795	Y	GAFTGA	GAF-PAS	HTH_8
CLSPOx_17460	*Clostridium sporogenes* strain DSM 795	Y	GAYTGA	PRD-EIIA-PRD	MarR
CLSPOx_18020	*Clostridium sporogenes* strain DSM 795	Y	GAFTGA	PTS_HPr-PAS-PAS	HTH_8
BGI42_00415	*Clostridium taeniosporum* strain 1/k	Y	GSFTGA	+	+
BGI42_03040	*Clostridium taeniosporum* strain 1/k	Y	GAFSGA	GAF-PAS	HTH_8

BGI42_03185	*Clostridium taeniosporum* strain 1/k	Y	GAFTGA	GAF-PAS	HTH_8
BGI42_06320	*Clostridium taeniosporum* strain 1/k	Y	GAFTGA	PRD-EIIA-PRD	+
BGI42_07600	*Clostridium taeniosporum* strain 1/k	Y	GAFTGA	GAF-PAS	HTH_8
BGI42_12770	*Clostridium taeniosporum* strain 1/k	Y	GTFTGG	GAF-PAS	HTH_8
CTC00072	*Clostridium tetani* E88	Y	GAFTGA	GAF-PAS	HTH_8
CTC00556	*Clostridium tetani* E88	Y	GAFTGA	GAF-PAS	+
CTC00707	*Clostridium tetani* E88	Y	GAFTGA	PAS-PAS	HTH_8
CTC01797	*Clostridium tetani* E88	N	GAFTGA	RR	HTH_8
CTC02322	*Clostridium tetani* E88	N	GAFTGA	RR	HTH_8
CTC02531	*Clostridium tetani* E88	Y	GSFTGA	ACT-ACT-PAS	+
CTK_RS01710	*Clostridium tyrobutyricum* strain KCTC 5387	Y	GSFTGA	GAF-PAS	HTH_8
CTK_RS03315	*Clostridium tyrobutyricum* strain KCTC 5387	Y	GAFTGA	PAS	HTH_8
CTK_RS03630	*Clostridium tyrobutyricum* strain KCTC 5387	Y	GAFTGA	PAS	HTH_8
CTK_RS06310	*Clostridium tyrobutyricum* strain KCTC 5387	Y	GSFTGA	GAF-PAS	HTH_8
CTK_RS07815	*Clostridium tyrobutyricum* strain KCTC 5387	Y	GAFTGA	GAF-PAS	HTH_8
CTK_RS07975	*Clostridium tyrobutyricum* strain KCTC 5387	Y	GSFTGA	PAS-PAS	HTH_8
CTK_RS08200	*Clostridium tyrobutyricum* strain KCTC 5387	Y	GSFTGA	GAF-PAS	HTH_8
CTK_RS10060	*Clostridium tyrobutyricum* strain KCTC 5387	Y	GSFTGA	PRD-EIIA-PRD	+
CTK_RS11890	*Clostridium tyrobutyricum* strain KCTC 5387	Y	GAFTGA	PAS	HTH_8
CTK_RS13650	*Clostridium tyrobutyricum* strain KCTC 5387	Y	GTFTGA	GAF-PAS	HTH_8
CTK_RS13810	*Clostridium tyrobutyricum* strain KCTC 5387	Y	GAFTGA	PTS_HPr-PAS-PAS	+
Gferi_00590	*Geosporobacter ferrireducens* strain IRF9	Y	GAFTGA	CBS-CBS-PAS	HTH_8
Gferi_03315	*Geosporobacter ferrireducens* strain IRF9	Y	GAFTGA	PAS	HTH_8
Gferi_03380	*Geosporobacter ferrireducens* strain IRF9	Y	GAFTGA	CBS-CBS-PAS	+
Gferi_06260	*Geosporobacter ferrireducens* strain IRF9	Y	GAYTGA	+	HTH_8
Gferi_06535	*Geosporobacter ferrireducens* strain IRF9	Y	GSFTGA	+	HTH_8
Gferi_07205	*Geosporobacter ferrireducens* strain IRF9	Y	GAFTGA	PAS	HTH_8
Gferi_07830	*Geosporobacter ferrireducens* strain IRF9	Y	GAFTGA	PAS	HTH_8
Gferi_08345	*Geosporobacter ferrireducens* strain IRF9	Y	GAFTGA	PrpR_N-PAS	HTH_8
Gferi_10145	*Geosporobacter ferrireducens* strain IRF9	Y	GAFTGA	CBS-CBS-PAS	HTH_8
Gferi_10195	*Geosporobacter ferrireducens* strain IRF9	Y	GAFTGA	PAS	HTH_8
Gferi_11160	*Geosporobacter ferrireducens* strain IRF9	Y	GAFTGA	PAS	HTH_8
Gferi_11540	*Geosporobacter ferrireducens* strain IRF9	Y	GSFTGA	PAS	HTH_8
Gferi_11715	*Geosporobacter ferrireducens* strain IRF9	Y	GAFTGA	PAS-PAS	HTH_8
Gferi_13025	*Geosporobacter ferrireducens* strain IRF9	Y	GAFTGA	PAS	+
Gferi_13100	*Geosporobacter ferrireducens* strain IRF9	Y	GAFTGA	PTS_HPr-PAS-PAS	+
Gferi_13975	*Geosporobacter ferrireducens* strain IRF9	Y	GAFTGA	PAS	+
Gferi_16065	*Geosporobacter ferrireducens* strain IRF9	Y	GAFTGA	PAS-PAS	HTH_12
Gferi_16135	*Geosporobacter ferrireducens* strain IRF9	N	GSFTGA	RR	HTH_8
Gferi_16225	*Geosporobacter ferrireducens* strain IRF9	Y	GSFTGA	PAS	HTH_8
Gferi_18025	*Geosporobacter ferrireducens* strain IRF9	Y	GAFTGA	PTS_HPr-PAS-PAS	+
Gferi_20755	*Geosporobacter ferrireducens* strain IRF9	Y	GAFTGA	GAF-PAS	HTH_8
Gferi_24870	*Geosporobacter ferrireducens* strain IRF9	Y	GAFTGA	GAF-PAS	HTH_8
Gferi_25640	*Geosporobacter ferrireducens* strain IRF9	Y	GAFTGA	PRD-EIIA	+
CFE_0246	*Carboxydocella thermautotrophica* strain 019	Y	GAFTGA	ACT-PAS	HTH_8
CFE_0320	*Carboxydocella thermautotrophica* strain 019	N	GAFTGA	RR	HTH_8
CFE_0576	*Carboxydocella thermautotrophica* strain 019	Y	GSFTGA	PAS	HTH_8
CFE_0587	*Carboxydocella thermautotrophica* strain 019	Y	GAFTGA	PAS-PAS	HTH_8
CFE_1832	*Carboxydocella thermautotrophica* strain 019	N	GAFTGA	RR	HTH_8
CFE_2270	*Carboxydocella thermautotrophica* strain 019	Y	GAFTGA	PAS-PAS	HTH_8
CFE_2341	*Carboxydocella thermautotrophica* strain 019	Y	GAFTGA	GAF-PAS	HTH_8
CFE_2394	*Carboxydocella thermautotrophica* strain 019	Y	GAFTGA	CBS-PAS	HTH_8
CFE_2753	*Carboxydocella thermautotrophica* strain 019	Y	GAFTGA	Fer4-Fe_hyd_lg_C-FeS	HTH_8
DOZ58_00495	*Acetobacterium* sp. KB-1	Y	GAYTGA	GAF-PAS	HTH_8
DOZ58_00560	*Acetobacterium* sp. KB-1	Y	GSFTGA	PAS	HTH_8
DOZ58_11430	*Acetobacterium* sp. KB-1	Y	GAFTGA	GAF-PAS	HTH_8
DOZ58_14700	*Acetobacterium* sp. KB-1	Y	YAFTGA	+	+
DOZ58_15390	*Acetobacterium* sp. KB-1	Y	GAFTGA	PTS_HPr-PAS-PAS	+
Awo_c01730	*Acetobacterium woodii* DSM 1030	Y	GAFTGA	GAF-PAS	HTH_8
Awo_c08990	*Acetobacterium woodii* DSM 1030	Y	GAFTGA	PTS_HPr-PAS-PAS	+

Awo_c22970	*Acetobacterium woodii* DSM 1030	Y	GSFTGA	PAS	HTH_8
Awo_c27090	*Acetobacterium woodii* DSM 1030	Y	GAYTGA	GAF-PAS	HTH_8
Awo_c27950	*Acetobacterium woodii* DSM 1030	Y	GSFTGA	PAS	HTH_8
Awo_c33690	*Acetobacterium woodii* DSM 1030	Y	GAFTGA	V4R-PAS	HTH_8
ELI_0117	*Eubacterium limosum* KIST612	Y	GAFTGA	PAS	+
ELI_0346	*Eubacterium limosum* KIST612	Y	GAFTGA	PAS	HTH_23
ELI_0892	*Eubacterium limosum* KIST612	Y	GAFTGA	PAS	+
ELI_2713	*Eubacterium limosum* KIST612	Y	GSFTGA	PAS	HTH_8
ELI_4229	*Eubacterium limosum* KIST612	Y	GAFTGA	PAS	HTH_28
ELI_4296	*Eubacterium limosum* KIST612	Y	GAFTGA	PTS_HPr-PAS-PAS	+
ADJ67_00760	*Eubacterium sulci* ATCC 35585	Y	GGFTGA	PAS	HTH_8
ADJ67_01920	*Eubacterium sulci* ATCC 35585	N	GAYTGA	RR	HTH_8
ADJ67_03330	*Eubacterium sulci* ATCC 35585	Y	GAFTGA	+	HTH_8
HM1_0841	*Heliobacterium modesticaldum* Ice1	Y	GAFTGA	PTS_HPr-PAS-PAS	HTH_8
HM1_1289	*Heliobacterium modesticaldum* Ice1	N	GAFTGA	RR	HTH_8
CGC65_00195	*[Clostridium] bolteae* strain ATCC BAA-613	Y	GAFTGA	0	HTH_8
CGC65_01700	*[Clostridium] bolteae* strain ATCC BAA-613	Y	GAFTGT	PrpR_N-PAS	HTH_8
CGC65_22175	*[Clostridium] bolteae* strain ATCC BAA-613	Y	GAFTGA	PRD-EIIA	+
CGC65_23770	*[Clostridium] bolteae* strain ATCC BAA-613	Y	GAFTGA	+	HTH_8
CGC65_24710	*[Clostridium] bolteae* strain ATCC BAA-613	Y	GAFTGA	PAS	+
CGC65_25445	*[Clostridium] bolteae* strain ATCC BAA-613	Y	GSYTGA	PAS	HTH_8
A4V08_09290	*Lachnoclostridium* sp. YL32	Y	GAFTGA	PRD-EIIA	+
A4V08_10965	*Lachnoclostridium* sp. YL32	Y	GAFTGS	PrpR_N-PAS	HTH_8
A4V08_16460	*Lachnoclostridium* sp. YL32	Y	GAFTGA	0	HTH_8
A4V08_24850	*Lachnoclostridium* sp. YL32	Y	GAFTGA	PrpR_N-PAS	HTH_8
A4V08_24935	*Lachnoclostridium* sp. YL32	Y	GAFSGA	PrpR_N-PAS	HTH_8
OBV_04720	*Oscillibacter valericigenes* Sjm18-20	Y	GAFTGA	PAS	HTH_8
OBV_07670	*Oscillibacter valericigenes* Sjm18-20	N	GAFTGA	RR	HTH_8
OBV_33450	*Oscillibacter valericigenes* Sjm18-20	Y	GAFTGA	PAS	HTH_8
OBV_40620	*Oscillibacter valericigenes* Sjm18-20	Y	GAFTGA	PAS	HTH_8
DEHRE_10595	*Dehalobacter restrictus* DSM 9455	Y	GSFTGA	GAF	HTH_8
DEHRE_12585	*Dehalobacter restrictus* DSM 9455	Y	GAFTGA	+	HTH_8
DCF50_p1771	*Dehalobacter* sp. CF	Y	GAFTGA	+	HTH_8
DHBDCA_p1762	*Dehalobacter* sp. DCA	Y	GAFTGA	+	HTH_8
CEQ75_RS00170	*Dehalobacterium formicoaceticum* strain DMC	Y	GSFSGA	PAS	HTH_8
CEQ75_RS02500	*Dehalobacterium formicoaceticum* strain DMC	Y	GAFTGA	+	HTH_8
CEQ75_RS06330	*Dehalobacterium formicoaceticum* strain DMC	N	GAFTGA	RR	HTH_8
CEQ75_RS07795	*Dehalobacterium formicoaceticum* strain DMC	Y	GAFTGA	PAS	HTH_8
CEQ75_RS08750	*Dehalobacterium formicoaceticum* strain DMC	Y	GAFTGA	PAS	HTH_8
CEQ75_RS09890	*Dehalobacterium formicoaceticum* strain DMC	N	GAFTGA	RR	HTH_8
CEQ75_RS10715	*Dehalobacterium formicoaceticum* strain DMC	Y	GAFTGA	PAS	+
CEQ75_RS10785	*Dehalobacterium formicoaceticum* strain DMC	N	GAFTGA	RR	HTH_8
CEQ75_RS14525	*Dehalobacterium formicoaceticum* strain DMC	Y	GAFTGA	PAS	HTH_23
CEQ75_RS16060	*Dehalobacterium formicoaceticum* strain DMC	N	GAFTGA	RR	HTH_8
CEQ75_RS17735	*Dehalobacterium formicoaceticum* strain DMC	Y	GAFTGA	PAS	+
Dhaf_0198	*Desulfitobacterium hafniense* DCB-2	N	GAFTGA	RR	HTH_8
Dhaf_0233	*Desulfitobacterium hafniense* DCB-2	Y	GSFTGA	+	HTH_8
Dhaf_0306	*Desulfitobacterium hafniense* DCB-2	Y	GAFTGA	PAS	HTH_8
Dhaf_0365	*Desulfitobacterium hafniense* DCB-2	Y	GAFSGA	PAS	+
Dhaf_0464	*Desulfitobacterium hafniense* DCB-2	Y	GSFTGA	+	HTH_8
Dhaf_0515	*Desulfitobacterium hafniense* DCB-2	Y	GAFTGS	GAF-PAS	HTH_8
Dhaf_1966	*Desulfitobacterium hafniense* DCB-2	Y	GAFTGA	PrpR_N-PAS	HTH_8
Dhaf_2047	*Desulfitobacterium hafniense* DCB-2	Y	GAFTGA	+	HTH_8
Dhaf_2373	*Desulfitobacterium hafniense* DCB-2	Y	GSFTGA	PAS-PAS	HTH_8
Dhaf_2509	*Desulfitobacterium hafniense* DCB-2	Y	GSFTGA	PAS	HTH_8
Dhaf_3148	*Desulfitobacterium hafniense* DCB-2	Y	GAFTGA	GAF-PAS	HTH_8
Dhaf_3656	*Desulfitobacterium hafniense* DCB-2	Y	GAFTGA	PrpR_N-PAS	+
Dhaf_4608	*Desulfitobacterium hafniense* DCB-2	Y	GAFTGA	PAS-PAS	HTH_8
Dhaf_4639	*Desulfitobacterium hafniense* DCB-2	Y	GSFTGA	PAS-PAS	HTH_8
Dhaf_4674	*Desulfitobacterium hafniense* DCB-2	N	GAFTGA	RR	HTH_8

Dhaf_4779	*Desulfitobacterium hafniense* DCB-2	N	GAFTGA	RR	HTH_8
Dhaf_4873	*Desulfitobacterium hafniense* DCB-2	Y	GAFTGA	PAS	HTH_23
Desor_0442	*Desulfosporosinus orientis* DSM 765	Y	GAFTGA	+	HTH_8
Desor_0568	*Desulfosporosinus orientis* DSM 765	N	GAFTGA	RR	HTH_8
Desor_0581	*Desulfosporosinus orientis* DSM 765	Y	GTFTGA	GAF-PAS	HTH_8
Desor_0629	*Desulfosporosinus orientis* DSM 765	N	GAFTGA	RR	HTH_8
Desor_0838	*Desulfosporosinus orientis* DSM 765	Y	GAFTGA	PAS	HTH_8
Desor_0846	*Desulfosporosinus orientis* DSM 765	Y	GAFTGA	PAS	HTH_8
Desor_0865	*Desulfosporosinus orientis* DSM 765	Y	GAFTGA	PAS	HTH_8
Desor_1053	*Desulfosporosinus orientis* DSM 765	Y	GAFTGA	PAS-PAS	HTH_8
Desor_1123	*Desulfosporosinus orientis* DSM 765	Y	GAFTGA	PAS	HTH_23
Desor_1150	*Desulfosporosinus orientis* DSM 765	Y	GAFTGA	CBS-PAS	+
Desor_1252	*Desulfosporosinus orientis* DSM 765	N	GAFTGA	RR	HTH_8
Desor_1631	*Desulfosporosinus orientis* DSM 765	Y	GAFTGA	PAS	+
Desor_1673	*Desulfosporosinus orientis* DSM 765	Y	GAFTGA	PrpR_N	HTH_8
Desor_1689	*Desulfosporosinus orientis* DSM 765	Y	GAFTGA	PAS	HTH_8
Desor_1716	*Desulfosporosinus orientis* DSM 765	Y	GAFTGA	PAS	+
Desor_1770	*Desulfosporosinus orientis* DSM 765	Y	GAFTGA	PAS	HTH_8
Desor_1771	*Desulfosporosinus orientis* DSM 765	Y	GAFTGA	PAS	HTH_8
Desor_2532	*Desulfosporosinus orientis* DSM 765	Y	GAFTGA	GAF-PAS	HTH_8
Desor_2546	*Desulfosporosinus orientis* DSM 765	Y	GAFTGA	PAS	HTH_8
Desor_2571	*Desulfosporosinus orientis* DSM 765	Y	GAFTGA	CBS-CBS-PAS	HTH_8
Desor_2619	*Desulfosporosinus orientis* DSM 765	Y	GAFTGA	PAS-PAS	HTH_8
Desor_2999	*Desulfosporosinus orientis* DSM 765	Y	GAFTGA	PAS	HTH_8
Desor_3031	*Desulfosporosinus orientis* DSM 765	Y	GAFTGA	PrpR_N-PAS	HTH_8
Desor_3044	*Desulfosporosinus orientis* DSM 765	Y	GAFTGA	PAS	HTH_8
Desor_3054	*Desulfosporosinus orientis* DSM 765	Y	GAFTGA	PAS	HTH_8
Desor_3069	*Desulfosporosinus orientis* DSM 765	Y	GAFTGA	PAS	HTH_8
Desor_3516	*Desulfosporosinus orientis* DSM 765	Y	GAFTGA	CBS-CBS-PAS-PAS	HTH_8
Desor_3809	*Desulfosporosinus orientis* DSM 765	N	GAFTGA	RR	HTH_8
Desor_3917	*Desulfosporosinus orientis* DSM 765	Y	GAFTGA	PAS-PAS	HTH_8
Desor_4317	*Desulfosporosinus orientis* DSM 765	N	GAFTGA	RR	HTH_8
Desor_4336	*Desulfosporosinus orientis* DSM 765	Y	GAFTGA	PAS-PAS	HTH_8
Desor_4337	*Desulfosporosinus orientis* DSM 765	Y	GAFTGA	GAF-PAS	HTH_8
Desor_4401	*Desulfosporosinus orientis* DSM 765	Y	GAFTGA	PAS-PAS	HTH_8
Desor_4405	*Desulfosporosinus orientis* DSM 765	Y	GAFTGA	PAS	HTH_8
Desor_4754	*Desulfosporosinus orientis* DSM 765	N	GAFTGA	RR	HTH_8
B0537_00100	*Desulfotomaculum ferrireducens* strain GSS09	Y	GAFTGA	ACT-PAS	+
B0537_03740	*Desulfotomaculum ferrireducens* strain GSS09	Y	GAFTGA	ACT-PAS	HTH_8
B0537_06210	*Desulfotomaculum ferrireducens* strain GSS09	N	GAFTGA	RR	HTH_8
B0537_09255	*Desulfotomaculum ferrireducens* strain GSS09	Y	GAFTGA	PAS	HTH_8
B0537_09895	*Desulfotomaculum ferrireducens* strain GSS09	N	GAFTGA	RR	HTH_8
B0537_11715	*Desulfotomaculum ferrireducens* strain GSS09	N	GSFTGA	RR	HTH_8
Dred_0258	*Desulfotomaculum reducens* MI-1	Y	GAFTGA	GAF-PAS	HTH_8
Dred_1312	*Desulfotomaculum reducens* MI-1	N	GAFTGA	RR	HTH_8
Dred_1494	*Desulfotomaculum reducens* MI-1	Y	GAFTGA	PAS	HTH_8
Dred_1510	*Desulfotomaculum reducens* MI-1	N	GAFTGA	RR	HTH_8
Dred_1719	*Desulfotomaculum reducens* MI-1	Y	GAFTGA	CBS-PAS	HTH_8
Dred_1725	*Desulfotomaculum reducens* MI-1	Y	GAFTGA	CBS-CBS-PAS	HTH_8
Dred_1763	*Desulfotomaculum reducens* MI-1	Y	GAFTGA	CBS-CBS-PAS-PAS	+
Dred_1776	*Desulfotomaculum reducens* MI-1	Y	GAFTGA	CBS-PAS-PAS	HTH_38
Dred_2001	*Desulfotomaculum reducens* MI-1	N	GAFTGA	RR	HTH_8
Dred_2835	*Desulfotomaculum reducens* MI-1	Y	GAFTGA	CBS-CBS-PAS	+
Desru_0081	*Desulfotomaculum ruminis* DSM 2154	Y	GAFSGA	PAS	HTH_8
Desru_1385	*Desulfotomaculum ruminis* DSM 2154	N	GSFTGA	RR	HTH_8
Desru_2255	*Desulfotomaculum ruminis* DSM 2154	Y	GAFTGA	ACT-PAS	+
Desru_2284	*Desulfotomaculum ruminis* DSM 2154	Y	GAFTGA	CBS-CBS-PAS	HTH_8
Desru_2290	*Desulfotomaculum ruminis* DSM 2154	Y	GAFTGA	PAS	HTH_8
Desru_2482	*Desulfotomaculum ruminis* DSM 2154	Y	GAFTGA	CBS-CBS-PAS	+
Desru_2717	*Desulfotomaculum ruminis* DSM 2154	N	GAFTGA	RR	HTH_8

Desru_3359	*Desulfotomaculum ruminis* DSM 2154	Y	GAFTGA	ACT-PAS	HTH_8
Desru_3509	*Desulfotomaculum ruminis* DSM 2154	Y	GAFTGA	GAF-PAS	HTH_8
PTH_0605	*Pelotomaculum thermopropionicum* SI	N	GAFTGA	RR	HTH_8
PTH_0678	*Pelotomaculum thermopropionicum* SI	N	GAFTGA	RR	HTH_8
PTH_0844	*Pelotomaculum thermopropionicum* SI	Y	GAFSGA	GAF-PAS	HTH_8
PTH_2258	*Pelotomaculum thermopropionicum* SI	N	GAFTGA	RR	HTH_8
PTH_2438	*Pelotomaculum thermopropionicum* SI	Y	GAFTGA	+	HTH_8
PTH_2639	*Pelotomaculum thermopropionicum* SI	N	GAFTGA	RR	HTH_8
PTH_2900	*Pelotomaculum thermopropionicum* SI	Y	GAFTGA	GAF-PAS	HTH_8
TherJR_0985	*Thermincola potens* JR	Y	GAFTGA	GAF-PAS	HTH_8
TherJR_1684	*Thermincola potens* JR	Y	GAFTGA	PAS-PAS	HTH_8
TherJR_2500	*Thermincola potens* JR	Y	GAFTGA	GAF-PAS	HTH_8
TherJR_2992	*Thermincola potens* JR	Y	GAFTGA	Fer4-Fe_hyd_lg_C-FeS	HTH_8
CD0040	*Clostridioides difficile* 630	Y	GAFTGA	PRD-EIIA-PRD	+
CD0167	*Clostridioides difficile* 630	Y	GTFTGG	PrpR_N-PAS	+
CD0283	*Clostridioides difficile* 630	Y	GAFTGA	PRD-EIIA-PRD	+
CD0402	*Clostridioides difficile* 630	Y	GSFTGA	PAS-PAS	HTH_8
CD0441	*Clostridioides difficile* 630	Y	GGFTGA	PAS	HTH_8
CD0516	*Clostridioides difficile* 630	Y	GSFTGA	PRD-EIIA-PRD	+
CD0806	*Clostridioides difficile* 630	Y	GAFTGA	PAS	HTH_8
CD1186	*Clostridioides difficile* 630	Y	GSFTGA	PrpR_N-PAS	+
CD1412	*Clostridioides difficile* 630	Y	GAFTGA	PAS	+
CD1739	*Clostridioides difficile* 630	N	GSFTGA	RR	HTH_8
CD2092	*Clostridioides difficile* 630	Y	GAFSGA	0	HTH_8
CD2283	*Clostridioides difficile* 630	Y	GAFTGA	PRD-EIIA-PRD	+
CD2328	*Clostridioides difficile* 630	Y	GAYTGA	PRD-EIIA-PRD	+
CD2383	*Clostridioides difficile* 630	Y	GAFTGA	PrpR_N-PAS	+
CD2700	*Clostridioides difficile* 630	Y	GSFTGA	PrpR_N-PAS	+
CD2732	*Clostridioides difficile* 630	Y	GAFTGA	PrpR_N-PAS	+
CD2863	*Clostridioides difficile* 630	Y	GSFTGA	PrpR_N-PAS	+
CD2869	*Clostridioides difficile* 630	Y	GSFTGA	PrpR_N-PAS	HTH_8
CD3094	*Clostridioides difficile* 630	Y	GAFTGA	PrpR_N-PAS	+
CD3186	*Clostridioides difficile* 630	Y	GAFSGA	GAF-PAS	+
CD3233	*Clostridioides difficile* 630	Y	GAFTGA	GAF-PAS	HTH_8
CD3245	*Clostridioides difficile* 630	Y	GAFTGA	CBS-CBS-PAS	HTH_8
CD3280	*Clostridioides difficile* 630	Y	GAYTGA	PRD-EIIA-PRD	+
CRIB_177	*Romboutsia ilealis* strain CRIB	Y	GAFTGA	GAF-PAS	HTH_8
FRIFI_0160	*Romboutsia* sp. Frifi	Y	GAFTGA	GAF-PAS	HTH_8
FRIFI_0665	*Romboutsia* sp. Frifi	Y	GAFTGA	PrpR_N-PAS	HTH_8
FRIFI_0793	*Romboutsia* sp. Frifi	Y	GGFTGA	PAS	HTH_8
FRIFI_0838	*Romboutsia* sp. Frifi	Y	GAFTGA	+	HTH_12
A4U99_00470	*Flavonifractor plautii* strain YL31	Y	GAFTGA	PAS	+
A4U99_00560	*Flavonifractor plautii* strain YL31	Y	GAFTGA	PAS	HTH_8
A4U99_06810	*Flavonifractor plautii* strain YL31	Y	GSFTGA	PAS	HTH_8
A4U99_11905	*Flavonifractor plautii* strain YL31	Y	GAFTGA	PAS	+
A4U99_12965	*Flavonifractor plautii* strain YL31	Y	GSYTGA	+	HTH_8
A4U99_15395	*Flavonifractor plautii* strain YL31	Y	GAFTGA	PAS-PAS	HTH_8
A4U99_15435	*Flavonifractor plautii* strain YL31	Y	GAFTGA	PAS	Rrf2
A4U99_15525	*Flavonifractor plautii* strain YL31	Y	GAFSGA	PAS	HTH_8
A4U99_15605	*Flavonifractor plautii* strain YL31	Y	GAFTGA	PAS	HTH_8
STH1275	*Symbiobacterium thermophilum* IAM 14863	Y	GAFTGA	PRD-EIIA-PRD	TrmB
STH1831	*Symbiobacterium thermophilum* IAM 14863	Y	GAFTGA	PAS-PAS	HTH_8
STH2087	*Symbiobacterium thermophilum* IAM 14863	N	GAFTGA	RR	HTH_8
STH3299	*Symbiobacterium thermophilum* IAM 14863	N	GAFTGG	RR	HTH_8
STH644	*Symbiobacterium thermophilum* IAM 14863	Y	GAFTGA	PAS	HTH_8
STH701	*Symbiobacterium thermophilum* IAM 14863	Y	GSFTGA	PAS	+
Swol_0306	*Syntrophomonas wolfei* str. Goettingen	Y	GAFTGA	PAS-PAS	HTH_8
Swol_0437	*Syntrophomonas wolfei* str. Goettingen	Y	GAYTGA	PAS	HTH_8
Swol_0792	*Syntrophomonas wolfei* str. Goettingen	Y	GAFTGA	PAS-PAS	HTH_8
Swol_1487	*Syntrophomonas wolfei* str. Goettingen	Y	GAFTGA	CBS-CBS-PAS-PAS	+

Swol_1699	*Syntrophomonas wolfei* str. Goettingen	Y	GAFTGA	GAF-PAS	HTH_8
Swol_2029	*Syntrophomonas wolfei* str. Goettingen	Y	GAFTGA	PAS-PAS	HTH_8
Slip_0224	*Syntrophothermus lipocalidus* DSM 12680	Y	GAFTGA	+	HTH_8
Slip_0282	*Syntrophothermus lipocalidus* DSM 12680	Y	GAFTGA	CBS-CBS-PAS-PAS	HTH_38
Slip_0489	*Syntrophothermus lipocalidus* DSM 12680	Y	GAFTGA	PAS-PAS	HTH_8
Slip_0504	*Syntrophothermus lipocalidus* DSM 12680	Y	GAFTGA	PAS	HTH_8
Slip_1990	*Syntrophothermus lipocalidus* DSM 12680	Y	GAYTGA	PAS	HTH_8
Slip_2080	*Syntrophothermus lipocalidus* DSM 12680	Y	GAFTGA	GAF-PAS	HTH_8
IB211_00322c	*Intestinimonas butyriciproducens* strain af211	Y	GAFTGA	PAS-PAS	+
IB211_01400c	*Intestinimonas butyriciproducens* strain af211	Y	GAFTGA	PAS	HTH_8
IB211_01464c	*Intestinimonas butyriciproducens* strain af211	Y	GAFTGA	+	HTH_8
IB211_02571	*Intestinimonas butyriciproducens* strain af211	Y	GAFTGS	+	HTH_8
IB211_02669	*Intestinimonas butyriciproducens* strain af211	Y	GAFTGA	PAS	HTH_8
IB211_02971c	*Intestinimonas butyriciproducens* strain af211	Y	GAFTGA	PAS	+
IB211_03204	*Intestinimonas butyriciproducens* strain af211	Y	GAFTGA	PAS	HTH_8

'+' denotes that a functional domain may present but have no significant sequence similarity with any annotated domain in Pfam database.

'0' denotes that a functional domain is absent.

Table S3 Reconstructed regulons of EBPs in *Clostridiales*

EBPs	Predicted function	Target operon	First gene ID of target operon	σ^{54} Promoter sequence	σ^{54} promoter position [a]	UAS	UAS position [a]
CBO1628	AcoR	*hyp-acoABXCL-lplA*	CBO1629	TTGGCATAATAGTTGCTTT	-74	ATAGGAATAGGTAAATCAAC	-247
CKL_2272	AcoR	*acoABCL*	CKL_2271	TTGGCATGGATATTGCTTT	-63	ATTGAAAACGTTTTTTCAAT	-141
Csca_RS10800	AcoR	*acoABCL--hyp-lplA*	Csca_RS10795	TTGGCACGTATCTTGCTAA	-50	ATTGGGATTGGAAAGTCAAC	-222
CTK_RS06310	AcoR	*acoABCL*	CTK_RS06315	TTGGCATAGGATTTGCTGA	-102	TTTGAAATCGTTTATTCAAT	-49
Cbei_2180	AdhR	*adhA*	Cbei_1722	TTGGCATAAATATTGCTTT	-81	TGTCCTAAAAGCATACA	-380
						TGTAATAAAACTGAACA	-362
						TGTATTTTTTTAATACA	-331
		adhA2	Cbei_2181	TTGGCATGAATATTGCTTT	-110	TGTCCTAAAATATTACG	-369
						TGTATTAAATTTATACT	-351
						TGTATTATTTTAGGACA	-335
NT01CX_0469	AdhR	*adhA-pyrk-aspB*	NT01CX_0468	TTGGCACGAAAGTTGCTTA	-88	TGTACTAAAATTGAACA	-226
						TGTATTGAAATTGAACA	-208
						TGTTCAAAAAAAGAACA	-174
CBO1642	AdhR	*adhA*	CBO1643	TTGGCATAATACTTGCTTT	-86	TGTATAGTTTTTATACA	-355
						TGTTTAATTTTTATACA	-388
						TGTATAAGAAATATACA	-406
CKL_0542	AdhR	*adhA*	CKL_0543	TTGGCATAGTACTTGCTGA	-92	TGTTGCAAAAAACAACA	-214
						TGTTGCTTTTTACAACA	-181
CLJU_c24870	AdhR	*adhA*	CLJU_c24880	CTGGCACAATACTTGCTTA	-111	TGTATATAAAAACAACA	-249
						TGTTGTTTTTTACAACA	-198
		adhA2	CLJU_c39950	TTGGTACGAATTTTGCTAA	-69	TGTTCTTTTTTTGAACA	-214
Cspa_c27820	AdhR	*adhA*	Cspa_c27830	TTGGCATGGAAGTTGCTAT	-71	TGTCCTAAAATAATACA	-346
						TGTATTATTTTAGGACA	-313
Csca_RS06870	AdhR	*adhA*	Csca_RS06865	TTGGAATACATTTTGCTTA	-132	TGTTTTATAATCTAACA	-336
						TGTCTTATAATCTAACA	-318
						TGTCTTATAATCTAACA	-300
		adhA2	Csca_RS14120	TTGGCATGGGTTTTGCTAA	-86	TTTATTTTGTTAAAACA	-125
CLSA_RS07870	AdhR	*adhA*	CLSA_RS07875	TTGGCACGGAAATTGCTAT	-78	TGTACTAAAATAGGACA	-220
						TGTCCTAAAATAGGACG	-202
						TGTCTTGTTTTAGTACA	-169
CLJU_c42510	AguQ	*aguDA*	CLJU_c42500	TTGGCACGAAGATTGCTTA	-203	TATTTCAATATGAAATG	-343
						TATGTCACTTTGACATG	-278
		aguQT	CLJU_c42510	TTGGTATAAATGTTGCTTA	-73	TATTTCAAATTGATATG	-163
						TATGTCAAATTGAGATG	-150
		aguBC	CLJU_c42560	TTGGCATGGATATTGCTAA	-95	TATGTCAAATAAACTTG	-270
						TATGTCATTTTGAAATA	-226
						TATCCCAAATTGAAATA	-173
Cbei_3816	AhcR	*atoE-hbd-cotX*	Cbei_3821	TTGGCATATGCTTTGCTTA	-83	TGTGCATAGTTTTGCACA	-290
CLJU_c20120/CLJU_c20200	AorR	*aor-moaD*	CLJU_c20210	TTGGCATGGTTTATGCTAT	-63	TGTTCCGAAACAGAACA	-160
						TGATCTGAAATGGAACA	-142
						TGCACCAAGATGGAACA	-121
		moaD2	CLJU_c20050	TTGGCACAATTTATGCTTT	-97	TGATCTTTATTTGAGCA	-317
						TGATCTTTATTTGAGCA	-276
						TGATCTTTATTTGGGCA	-235
		aor2	CLJU_c20110	TTGGCACAATTTATGCTCT	-64	TGATCCATATTGGAACG	-314
CLSPOx_09730	AorR	*aor*	CLSPOx_09725	TTGGCATAAAACTTGCTTA	-71	TGTACTAATATAGAACA	-397
						AGTTCCAAAAAAGAACA	-379
						TGTATCTAAATAGAACA	-346
CBO1937	AorR	*aor-moaD*	CBO1935	TTGGCATAAAACTTGCTTA	-72	TGTACTAATATAGAACA	-175
						AGTTCCAAAAAGGAACA	-157
						TGTATCTAAATAGAACA	-124
Cbei_1463	BldR	*butA*	Cbei_1464	TTGGCACGAAAATTGCTTA	-68	TGTATCAAAATGAGAAA	-254
						TGTATCAAAATGAGAAA	-226
						TGTATCGTTTTGATACA	-203
CLJU_c23230	BldR	*butA*	CLJU_c23220	TTGGTATGGCTTTTGCTTA	-95	TGTGTCATAATGGCACA	-150

NT01CX_0345	BldR	*butA*	NT01CX_0344	TTGGTATTATAATTGCTTA	-80	TGTACCTATATGATACA	-187
						TGTATCATATAGGTACA	-159
Cspa_c22060	BldR	*butA*	Cspa_c22070	TTGGCATAGATATTGCTCT	-66	TGTATCAAAATGAGAAT	-244
						TGTATCAAAATGATAAT	-216
						TGTATCATTTTGATACA	-192
CLSA_RS19605	BldR	*butA*	CLSA_RS19600	TTGGCATGGATTTTGCTAT	-67	TGTATCAAAATGAGAAT	-253
						TGTATCAAAATGAGAAA	-225
						TGTATCATTTTGATGCA	-202
CTK_RS07815	BldR	*butA*	CTK_RS07810	ATGGCATGATATTTGCTTA	-99	TGTGCCATATTAATACA	-180
						TGAGTCAATATGGCACA	-155
Csca_RS09730	BldR	*butA*	Csca_RS09725	TTGGCATGCTATTTGCTAA	-67	CCTGTCAATATGGTACA	-148
						TGTGTCAATATGATACA	-123
Cbei_2039	CrbR	*cotX-gntT*	Cbei_2040	TTGGCATCCTAATTGCTCT	-46	TGTATAAAAAAAATACA	-266
						TGTAAAGGAAATAAACA	-249
						TGTACAAAAAAAACACA	-183
		crt-bcd-etfAB-maoC	Cbei_2034	TTGGCATGCTTATTGCTAA	-255	TGTACAAAATATAGACA	-344
Csca_RS12550	CrbR	*cotX-gntT*	Csca_RS12545	TTGGCATGATTGTTGCTTT	-61	TGTAAAAAAAATGAACA	-271
						TGTAAAAAATATATACA	-254
						TGTACATTAAATATACA	-188
		crt-hbd-thl-maoC-bcd-etfAB	Csca_RS12585	TTGGTATGCTTCTTGCTGA	-41	TGTAAAAATTATATACA	-215
						TGTAAAATATATAAACA	-198
						TGTAAAAATATTATACA	-133
CLSPOx_16515	CrbR	*crt-hbd-thl-bcd-etfAB*	CLSPOx_16545	CTGGCACGAAAGTTGCTTC	-42	TGTAAAAAAGATAAACA	-278
						TGTAATAAATATATACA	-261
CBO3196	CrbR	*crt-hbd-thl-bcd-etfAB*	CBO3202	CTGGCATGAAAGTTGCTTC	-42	TGTTATTAAATAAAACA	-301
						TGTAAAAAGAATAAACA	-279
						TGTAATAAATATATACA	-262
						TGTATATAATATACACA	-180
CA_C3088	DhaQ	*ptsI*	CA_C3087	TTGGCATGATAATTGCTAT	-62	TTGATAAAAATAATCAA	-373
						ATGATAATTTTTATCAA	-341
						ATGATAAAAAGTATCAA	-243
CBO3439	DhaQ	*ptsI*	CBO3438	ATGGCATGGTTTTTGCTAA	-87	TTGATAAAAAATATCAA	-304
						TTGATAAAAAATATCAA	-201
CPF_2667	DhaQ	*ptsI*	CPF_2666	TTGGCACGCAAATTGCTGT	-118	TTGATAAAAAATATCAA	-307
						TTGATATTTTTTATCAC	-277
CTK_RS13810	DhaQ	*ptsI*	CTK_RS13805	TTGGCATGAAAATTGCTTT	-101	ATGCTAAAATATATCAA	-375
						TTGATATTTTTTATCAA	-344
						TTGATATTTTTTATCAT	-244
Cbei_2147	DhaQ	*dhaKLM*	Cbei_2148	GTGGCATGAGTATTGCTCT	-324	TTGGTAAAAAGTATCAT	-482
						TTGATACTTTTTACCAA	-449
CBO2889	DioR	*dpaL-pbuX-pyrC*	CBO2888	TTGGCACAAATATTGCTTA	-58	ATTTATCAAAGTGATAAGT	-298
		ygeW-ygfK-ssnA-ygeY	CBO2885	TTGGCATAAATATTGCTTA	-125	ATTTATCATAATGATAAGG	-282
						ATTTATCATTTTAATAATA	-195
						TATTATTAAAATGATAAAT	-212
		yqeC-ygfJ-yqeB-xdhC-hyp-fepDCB	CBO2890	TTGGCATATTTATTGCTTC	-57	ATTTATCAATAGGATAATT	-249
						ATTTATCATTTTAATAATT	-175
		hyp-xdhABC	CBO1283	TTGGTATAGTTTTTGCATC	-207	GTTTATCGTTTTGATAAAA	-367
						ATTTATCAAAATGATATAT	-387
CTC00072	DioR	*dpaL*	CTC00071	TTGGTATGTATATTGCTAT	-60	TATTATCAAAGTGATATTT	-217
						TATTATCATTTTGATAATA	-199
CD3186/CD2092	DioR	*dpaL-pyrC-argE*	CD2085	TTGGCATGTTAATTGCTTA	-64	AATTTTAAAAATGAGAATT	-227
		tdcF	CD3187	TTGGCACGTTAATTGCTTT	-174	ATTTCTAGATATGAGAAAA	-295
						AATTCTCAAAACAAGAATT	-327
		dpaL	CD3184	TTGGCACGGTAATTGCTTT	-85	AATTCTCGTATTGATAAAA	-232
						ATTTCCCAATATGAGAATT	-213
						ATTTCTCGAATTGATAATT	-247
		yqeB	CD3478	GTGGCATGTTAGTTGCTTA	-96	ATTTCTCGAATTGAGAATA	-185
						AATTATTGAATTGATAAAT	-202
		dioR	CD2092	ATGGCATTGTAATTGCTTA	-207	AATTTTAAAATTTATAAAA	-319
		pbuX	CD2091	TTGGCATTATAATTGCTTC	-94	AATTATAAAATTGATAAAA	-278

CLJU_c40410	DioR	*tdcF*	CLJU_c40370	TTGGCATGGATATTGCTCT	-79	AATTCTCAAGTTAAGATTT	-177
		hyp	CLJU_c40400	TTGGTATAGATATTGCTCT	-78	AATTCTTAAGTTGAGATTT	-176
CLSPOx_14815	DioR	*dpaL-pbuX-pyrC*	CLSPOx_14810	TTGGCACAAGTATTGCTTA	-59	ATTTATCAAAATGATAAAT	-300
						TTTTACCGTAATGATAATT	-241
						AATTATTATTTTGATAATA	-222
		ygeW-ygfK-ssnA-ygeY	CLSPOx_14795	TTGGCATAAATATTGCTTA	-125	ATTTATCAAAATGATAAGA	-284
						TATTATTAAAATGATAAAT	-214
						ATTTATCATTTTAATAATA	-197
		yqeC-ygfJ-yqeB-xdhC-hyp-fepDCB	CLSPOx_14820	TTGGCATATTTATTGCTTC	-59	ATTTATCAATAGGATAATT	-251
						TTTTATCATTTTAATAATT	-176
CLJU_c36950	GamR	*puuD*	CLJU_c36940	ATGGCATAAAAAATGCTTA	-62	TCATTGATATCATGCAATTT	-229
CD0167/CD1186/CD3094	GamR	*abgB-puuT*	CD0166	ATGGCATAATAATTGCTTA	-60	AAATTGAAACTATGCAATCA	-158
		hyp-puuDT	CD1187	TTGGCATACATATTGCTAA	-57	GAATTAGAAGTAAAAAATAC	-134
		puuD	CD3093	TTGGTATGCTACTTGCTCT	-63	ATATTACTCGTATTCAAAAA	-125
Csca_RS07205	GamR	*puuTD*	Csca_RS07200	TAGGCATAAAAATTGCTAG	-52	TTTTTGAAAATAAAAAATTT	-236
						ATTTTGCTTTGAATCAAAAT	-170
CLJU_c40670	GasR	*puuXP*	CLJU_c40680	TTGGTATAATTATTGCTTG	128	TATTGTCATAATGATTTCTCATTATGACAATA	-208
Csca_RS07205	GasR	*puuXP*	Csca_RS23005	GTGGCATGTTTATTGCTAT	125	TATTATTAAAATGACAATTCATTTTGACAATA	-206
CLJU_c32880	HiaR	*hiaL-hyp-hutH*	CLJU_c32870	CTGGCATGACTTTTGCTAT	-70	CTTTTTTAAAATCATAG	-161
CLJU_c24850	SadR	*sdh*	CLJU_c24860	CTGGCACAACATTTGCTTA	-96	TGTTGTAAAAAGCAACA	-219
Csca_RS05205	SadR	*sdh*	Csca_RS05210	TTGGCATTATTATTGCTGA	172	TGTATAAAAAAACAACA	-309
						TGTTGAAAAAAACAACA	-291
						TGTTGATAATAACAACA	-259
CAETHG_0552	SadR	*sdh*	CAETHG_0553	CTGGCACAACATTTGCTTA	-96	TGTTGTAAAAAGCAACA	-219
CTK_RS13650	SadR	*sdh*	CTK_RS13645	ATGGCATATATGTTGCTTA	-80	TGTTTAAAAAATACAACG	-241
CBO2009	CdsR1	*cdsB*	CBO2008	TTGGCATCCTTATTGCTAC	-57	ATTTGCAAAAGTAAGAAT	-296
						TTTTGCAAAGATGCAAAA	-276
						ATTTGCAAAAAATGCAAAA	-205
CLSPOx_10150	CdsR1	*cdsB*	CLSPOx_09895	TTGGCATGGTTTTTGCTCA	-72	ATTTGCAAAAGTGAGAAT	-274
						ATTTGCAATGATGCAAAA	-183
Csca_RS11550	CdsR1	*cdsB*	Csca_RS11545	ATGGCATGGTATTTGCTAG	-51	ATTGTCATTTTTGAGAAA	-304
						ATTCTTATTTTTGAGAAA	-161
CD3233	CdsR2	*cdsB*	CD3232	ATGGCATGTATTTTGCTAT	-116	CGTCTCAAAATGATATAGAATCTCATTTTGAGACT	-298
Cbei_3875/Cbei_4915	LevR	*levDEFG-sacC*	Cbei_3874	TTGGCATAGGAATTGCTAT	-68	TGTGTGTTAA-(14)-TTAATAAACT	-222
		levDEFG	Cbei_4914	TTGGCATGGGAATTGCTAG	-68	TGTGTGTTAAA-(23)-TGTGTCAAAAT	-224
						AATGTGTCAAA-(13)-TGTGTCAAATA	-192
						AGTGTGTCAAA-(11)-AGTGTCAAGGT	-170
Cspa_c55920	LevR	*levDEFG*	Cspa_c55910	TTGGCATGATAATTGCTTA	-65	TGTGTGTTAAA-(22)-AGTGTCAAAAA	-185
						AGTGTGTCAAA-(11)-AGTGTCAAGAT	-133
CLSA_RS09985/CLSA_RS21470	LevR	*levDEFG-sacC*	CLSA_RS09990	TTGGCATGACAATTGCTAT	-71	TGTGTATTAAT-(11)-ATTAAGAAAAT	-227
						TGTGTCTTAAG-(9)-TTTAAAATTAA	-368
		levDE	CLSA_RS21475	TTGGCACACCATTTGCTAT	-68	TCCGTATCAAT-(11)-TTCATGAAAAT	-224
						AGTGTATTAAC-(15)-TTTATTTCACT	-151
CBO0028	MdeR	*mdeA*	CBO0029	TTGGTATATTATTTGCTTT	-57	TGTAAATAAATATTTACA	-169
						TGTAAAAAAATATTGCCA	-149
CLJU_c36300	MdeR	*metT*	CLJU_c36290	TTGGCATAATAAATGCTAA	-69	TGTAAAGAAATGTTTACA	-200
CLSPOx_00150	MdeR	*mdeA*	CLSPOx_00155	TTGGCATATTATTTGCTCA	-57	TGTAAATAAATATTTACA	-179
						TGTAAAAAAACATTGCCA	-159
NT01CX_0150	MdeR	*mdeA*	NT01CX_0149	TTGGCATGCAACTTGCTTA	-64	TGGCAATAATAATTTACA	-383
Csca_RS14075	MdeR	*mdeA*	Csca_RS14080	TTGGCACAAAATTTGCTTT	-64	TGTAAATAAAACTTTACA	-143
CTC02531	MdeR	*mdeA-metT*	CTC02530	TTGGAACGATTATTGCTTT	-60	TGGAAAGAATAATTTACA	-210
CLJU_c24140	MopR	*mopR*	CLJU_c24140	ATGGCATATATATTGCTTA	179	TAGTCAAAAAACGAACA	-279
						TGATCAAAAAGTGAACA	-245
		mop	CLJU_c24130	TTGGCATACTTATTGCTTA	127	TGACCGAAAAACGAACA	-246
						TAATCAAAAAATGAACA	-228
						TGATCTAAAAATGAGCA	-194
		hyp-moeA	CLJU_c17940	GTGGCATACATATTGCTTG	171	TAGTCAAAAAACGAACA	-272
						TGATCTAAAAATGAACA	-238
Cspa_c06870	MopR	*moeA-mop*	Cspa_c06880	TTGGCACGGAATTTGCTTG	-79	TATTCCAAAATAGAACA	-228
						TGTTCTGAAATGGAACA	-210

						TGTGTCAAAATGGAACA	-176
CD0441	OrdR	*ord-ortBA-oraSEF-orr-nhaC*	CD0442	TTGGCACGATTTATGCTTT	-62	GAGCAAAATATTTTGCTC	-185
Csca_RS00565	OrdR	*ortBA-oraSEF*	Csca_RS00570	ATGGCTTACTATTTGCTTT	-96	TAGCAAAATAATTTGTCA	-264
						TGTAAAAATATTTTGCAT	-244
						TAGCAAGAAATTTTGCTG	-165
CBO2490	PrdR	*prdC*	CBO2466	TTGGCATAAACATTGCTAT	-58	TGTCTAAATATTGACA	-231
						TGTCAAAAAAAAGACA	-200
		prdC2-prdA	CBO2482	TTGGCATAGTTTATGCTTT	-53	TGTCAAAAAAATAACG	-251
						TGTCAAAAAAACGACA	-164
CD3245	PrdR	*prdC*	CD3244	TTGGCATAGGAATTGCTTA	-176	TGTTGATTAATTGACA	-321
		prdABDE-prdE2-prdF	CD3247	TTGGCATAGAAATTGCTTT	-140	TGTCGATAAAAAGACG	-325
						TGTCTATTTTTTGACA	-308
						TGTCTAATAATTGACT	-238
CLSPOx_12930	PrdR	*prdC-hyp-prdA-hyp-prdB*	CLSPOx_12805	TTGGCATAAACATTGCTAT	-57	TGTCAAAAAAATAACG	-283
						TGTCAAAAAAATAACA	-196
		prdC2-hyp-prdA2-hyp-prdB2-hyp	CLSPOx_12885	TTGGCATAGTTTATGCTTA	-59	TGTCTAAAAGCTGACA	-233
						TGTCAAAAAAAAGACA	-202
		prdC3-hyp-prdA3-hyp-prdB3	CLSPOx_18850	TAGGCACAATAAATCCTAT	-234	TGTCAAAAAATGAAAT	-289
						TGTTGGATTTTTAACA	-206
Csca_RS19145	PrdR	*prdCA-hyp-prdBDEF-hyp*	Csca_RS19140	TTGGCACAATAAATGCTGC	-54	TGTCTAATTATTGACA	-256
		prdC2-prdA2-hyp-prdB2-prdD2-prdE2-prdF2	Csca_RS19090	TTGGCACGGAAATTGCTTA	170	TGTCAAATTATTGACA	-376
		prdC3-hyp-prdA3-prdB3	Csca_RS22005	TTGGCATATGAATTGCTAT	223	GGACAATTTTTTAACA	-349
CD1739	SarR	*grdGF*	CD1740	TTGGCATAGAAAATGCTTT	-82	TGAGAAAAAAACTTCTCA	-282
						TGAGAAAAAAATTTCTCA	-259
Cbei_2099	XanR	*abfD-nifU-hyp-ach-golB*	Cbei_2100	TTGGCACGCTTATTGCTTT	-62	GGTGCAAAAATGCAAC	-279
						GATGCAGAAGTGCAAC	-261
						GTTGCGTTTTTGCAAC	-171
CKL_3021	XanR	*abfD-nifU-ach-orfY-ach-sucD-hbd*	CKL_3020	TTGGCATGAAAGTTGCTAA	-69	GTTGCAATTATGCATA	-283
		acp-caiC-luxEC	CKL_3027	GTGGCATGATAATTGCTTT	-57	TATGCAAAATTGCAAC	-192
						GTTGCATTAATGCAAT	-123
CTK_RS03630	XanR	*acp-caiC-luxEC-hyp*	CTK_RS03610	GTGGCATGGTATTTGCTTT	-55	GATGCATATTTGCAAC	-205
						GTTGCATACATGCATC	-123
		abfD-nifU-ach-hyp	CTK_RS03710	TTGGCATCAGATTTGCTTG	-73	GTTGCAATTATGCAAT	-287
						GATGCAATAATGCAGC	-198
Cbei_3352	XcgR	*cotY-gntT-maoC*	Cbei_3351	TTGGCACGGATATTGCTTA	-105	TGTTCGTTCTTACGAACA	-287
						TGTACGGAAAATCGCACA	-245
						TGTACGGAATAACGAACA	-202
Cspa_c20830	XcgR	*cotY-gntT*	Cspa_c20850	TTGGCACGAGTCTTGCTTA	-96	TGTACGAATAAACGTACA	-280
						TGTATAGAAATCCGAACA	-238
						TGTACGGAAATCTATACA	-195
CTC00707	XcgR	*cotY-gntT-bcd-etfAB*	CTC00708	TTGGCATAATAATTGCTTT	-58	TGTAAGGTAACAAGTACA	-263
						TGTAAGGAAAACACTACA	-221
						TGTACGGAAATATAAACA	-179
CD2869	XduR	*kdgT-uxaA*	CD2870	TTGGCATAGTAATTGCTTT	-57	TGTTTAAAAATAATACA	-278
						TGTTTAATTTTAATACA	-236
CD1412	XhaQ	*rhaT*	CD1413	ATGGCATAGTTTTTGCTTA	-66	TAAGTTATATATAACTCA	-208
						TAAGTCTAAATCAACTTA	-176
CD0806	YctR	*crt-gntT-caiB-bcd-etfAB*	CD0800	TTGGCATAGTACTTGCTAT	-85	ATATCAAAAATGAGAC	-322
						TTCTCAAAAATGAGAC	-302
						TTCTCGAAAATGAGAC	-269
CBO0594	YpdR	*pepABC*	CBO1355	TTGGCATGGATTTTGCTTA	-70	TTTTCTCAATATGAGAGAA	-227
						AATTATCAAAATGATACAA	-200
						TTTTCTCAAAATGACACAA	-177
		fmdE-pepABC	CBO1674	TTGGCATATTAATTGCTAC	-226	ATGTATCAAAATGATAAAA	-332
						AAATATCAAAATGGAACAT	-315
						AAGTATCATTTTGAGAATA	-293
CLJU_c22680	YpdR	*pepABC*	CLJU_c22670	CTGGCATAAATATTGCTGA	-71	CTTTATCAGAATGAAACTA	-253
						TAATATCATTATGGAACAA	-234
						TAATATCATAATGATATTA	-213

		memP-metM	CLJU_c22700	TTGGCATAAAAATTGCTTT	-55	TATTATCATAATGATAAAA	-369
						CGGTATCGTAATGATAATA	-347
						TTTTATCATTACGATACCA	-319
		metM-memP-pepABC	CLJU_c22620	CTGGCATAAAATTTGCTGT	-71	TAGTTTCATAATGATAAAA	-165
						AAATCTCAAAGTGATACAA	-147
		fmdE-metM-hemABC	CLJU_c38060	TTGGCTTGAAAATTGCTTG	-51	TAGTCTCATAATGATGCTA	-248
						CTGTATCATTATAATAAAA	-207
						TATCATTATAATAAAATTA	-204
		hemABC	CLJU_c38000	TTGGTACAAAAATTGCTAA	176	TAGTTTTAAAATGATAAAA	-354
						TTTTATCAAAATGATATTG	-313
Csca_RS24105	YpdR	*pepABC-memP-hemABC*	Csca_RS02505	TTGGCATAAAAAATGCTTT	-78	CTGTTTCAATATGATAAAA	-322
						AAATATCATATTGAAACCG	-305
						TTGTATCTTTTTGATAAAA	-283
		metM	Csca_RS02405	GTGGCATGGAAATTGCTGG	-63	ATGTTTCAAAATGATAAAA	-169
						TATTATCATTTTGAAACTG	-142
						AATTATCATTATGATACAA	-119

[a] Positions of the 5' end of the σ^{54} promoter sites and UAS sites are given relative to the translational start.

Table S4. Primers used in this study

Protein overexpression and purification	
pET28a-*sigL*	5'-AGAGAGGGATCCATGAATTTGGATTATAACATG-3'<br>5'-AGAGAGGCGGCCGCTTATAATCTCTTCCTTGCAG-3'
Amplification of 200-bp DNA fragments from the upstream region of genes for EMSA	
Cbei_2034(*crt*)	5'-AGCCAGTGGCGATAAGGGTGTACAAAATATAGACA-3'<br>5'-AGCCAGTGGCGATAAGTCCTTAAATAGCAAATAGC-3'
Cbei_2040(*cotX*)	5'-AGCCAGTGGCGATAAGGGGGAAAATCTTTAAAAG-3'<br>5'-AGCCAGTGGCGATAAGTCATAATCCCTCCTCATACC-3'
CD3244(*prdA*)	5'-AGCCAGTGGCGATAAGAACATATTTTAAATAAATA-3'<br>5'-AGCCAGTGGCGATAAGCCCCTTAACTGAGAAATTAAG-3'
CD3247(*prdC*)	5'-AGCCAGTGGCGATAAGAATTGACTTTTTTTTGATGGG-3'<br>5'-AGCCAGTGGCGATAAGTTATAAGCTGTTCAAATAATG-3'
CD0395(*hadA*)	5'-AGCCAGTGGCGATAAGAGTGGCAAGATGCCACGATATG-3'<br>5'-AGCCAGTGGCGATAAGTCTGATGTCAAAGTTCCTAATC-3'
CLJU_c23220(*butA*)	5'-AGCCAGTGGCGATAAGCCAGAAAAGAGTAAGATTCTC-3'<br>5'-AGCCAGTGGCGATAAGAATTATCTCTCCTTTTTTATAATAG-3'
